# Docosahexaenoic Acid Consumption Impedes Early Interferon- and Chemokine-Related Gene Expression While Suppressing Silica-Triggered Flaring of Murine Lupus

**DOI:** 10.3389/fimmu.2019.02851

**Published:** 2019-12-13

**Authors:** Abby D. Benninghoff, Melissa A. Bates, Preeti S. Chauhan, Kathryn A. Wierenga, Kristen N. Gilley, Andrij Holian, Jack R. Harkema, James J. Pestka

**Affiliations:** ^1^Department of Animal, Dairy and Veterinary Sciences and The School of Veterinary Medicine, Utah State University, Logan, UT, United States; ^2^Department of Food Science and Human Nutrition, Michigan State University, East Lansing, MI, United States; ^3^Institute for Integrative Toxicology, Michigan State University, East Lansing, MI, United States; ^4^Department of Biochemistry and Molecular Biology, Michigan State University, East Lansing, MI, United States; ^5^Department of Biomedical and Pharmaceutical Sciences, Center for Environmental Health Sciences, University of Montana, Missoula, MT, United States; ^6^Department of Pathobiology and Diagnostic Investigation, Michigan State University, East Lansing, MI, United States; ^7^Department of Microbiology and Molecular Genetics, Michigan State University, East Lansing, MI, United States

**Keywords:** omega-3 polyunsaturated fatty acids, autoimmunity, nanostring, lung, kidney, systemic lupus erythematosus, silica, transcriptome

## Abstract

Exposure of lupus-prone female NZBWF1 mice to respirable crystalline silica (cSiO_2_), a known human autoimmune trigger, initiates loss of tolerance, rapid progression of autoimmunity, and early onset of glomerulonephritis. We have previously demonstrated that dietary supplementation with the ω-3 polyunsaturated fatty acid docosahexaenoic acid (DHA) suppresses autoimmune pathogenesis and nephritis in this unique model of lupus flaring. In this report, we utilized tissues from prior studies to test the hypothesis that DHA consumption interferes with upregulation of critical genes associated with cSiO_2_-triggered murine lupus. A NanoString nCounter platform targeting 770 immune-related genes was used to assess the effects cSiO_2_ on mRNA signatures over time in female NZBWF1 mice consuming control (CON) diets compared to mice fed diets containing DHA at an amount calorically equivalent to human consumption of 2 g per day (DHA low) or 5 g per day (DHA high). Experimental groups of mice were sacrificed: (1) 1 d after a single intranasal instillation of 1 mg cSiO_2_ or vehicle, (2) 1 d after four weekly single instillations of vehicle or 1 mg cSiO_2_, and (3) 1, 5, 9, and 13 weeks after four weekly single instillations of vehicle or 1 mg cSiO_2_. Genes associated with inflammation as well as innate and adaptive immunity were markedly upregulated in lungs of CON-fed mice 1 d after four weekly cSiO_2_ doses but were significantly suppressed in mice fed DHA high diets. Importantly, mRNA signatures in lungs of cSiO_2_-treated CON-fed mice over 13 weeks reflected progressive amplification of interferon (IFN)- and chemokine-related gene pathways. While these responses in the DHA low group were suppressed primarily at week 5, significant downregulation was observed at weeks 1, 5, 9, and 13 in mice fed the DHA high diet. At week 13, cSiO_2_ treatment of CON-fed mice affected 214 genes in kidney tissue associated with inflammation, innate/adaptive immunity, IFN, chemokines, and antigen processing, mostly by upregulation; however, feeding DHA dose-dependently suppressed these responses. Taken together, dietary DHA intake in lupus-prone mice impeded cSiO_2_-triggered mRNA signatures known to be involved in ectopic lymphoid tissue neogenesis, systemic autoimmunity, and glomerulonephritis.

## Introduction

Systemic lupus erythematosus (SLE) is a devastating multisystem autoimmune disease that primarily affects women of childbearing age and non-Caucasians (1, 2). SLE is initiated following breakdown of immune tolerance resulting from incompletely understood interactions between an individual's susceptibility genes and the environment. Early stage SLE involves a chronic autoimmune response, characterized by antibody production against self-antigens and the subsequent formation of immune complexes. The latter promote complement activation, cell death, chemokine/cytokine release, and mononuclear effector cell infiltration resulting in systemic inflammation and progressive organ damage that is often exacerbated by acute disease flares triggered by environmental stimuli. In the kidney, these responses can manifest as severe glomerulonephritis that often leads to end-stage renal failure. SLE is currently managed by decreasing disease symptoms in recently diagnosed persons and inhibiting further tissue damage in organs, such as the kidney, in long-term patients. Current therapies have multiple mechanisms of action including immunosuppression, lymphocyte depletion, and cytokine/chemokine neutralization. These approaches have serious limitations including unacceptable side effects, irreversible drug-induced organ damage, and high costs for new targeted monoclonal antibody/receptor therapies.

Murine models of SLE have been used to understand disease pathogenesis and show gradual accumulation of autoreactive B and T cells as well accumulation of autoantibodies followed by eventual onset of organ damage [reviewed in (3)]. Therefore, these models typify quiescent SLE prior to organ damage heralded by glomerulonephritis. However, flaring can be induced in these models and organ damage accelerated by injection of IFNα-expressing adenovirus (4–6), UV exposure (7, 8), and epidermal injury (9). Crystalline silica (cSiO_2_) is a respirable particle commonly encountered in occupations such as construction and mining that has been etiologically linked to SLE and other autoimmune diseases (10). Prior investigations in lupus-prone mice have demonstrated that airway exposure to cSiO_2_ rapidly accelerates the onset and progression of autoimmunity thus emulating flaring (11–14). We have determined that short-term cSiO_2_ instillation of female NZBWF1 mice triggers autoimmunity and glomerulonephritis 3 months earlier than vehicle-instilled controls (15, 16). Specifically, cSiO_2_ treatment mimics SLE flaring by initiating persistent sterile inflammation and cell death in the lung and initiating ectopic lymphoid structure (ELS) development. These tissue structures contain functional germinal centers that house B-cells, T-cells, follicular dendritic cells (FDC), and autoantibody-secreting plasma cells. Autoantibodies arising from ELS potentially form immune complexes with autoantigens formed in the lung following cSiO_2_ exposure that drive systemic autoimmunity and glomerulonephritis.

Recently, we utilized NanoString nCounter profiling to map dynamic transcriptome signature changes in cSiO_2_-exposed NZBWF1 mice (17). Dramatic upregulation mRNAs associated with interferon (IFN) activity, chemokine release, cytokine production, complement activation, and adhesion was observed in the lung during the first 2 months after cSiO_2_ treatment that corresponded closely with autoimmune pathogenesis. cSiO_2_ similarly induced robust changes in transcriptome signatures later in the kidney and in the spleen, to a lesser extent. Importantly, cSiO_2_-induced mRNA signatures consistent with the lung being central autoimmune nexus for initiating systemic autoimmunity and ultimately, glomerulonephritis.

Preclinical and clinical studies have shown that consumption of ω-3 polyunsaturated fatty acids (PUFAs), such as docosahexaenoic acid (C22:6 ω-3; DHA) and eicosapentaenoic acid (C20:5 ω-3; EPA), have the potential to prevent or treat many chronic inflammatory and autoimmune conditions [reviewed in (18)]. Western diets tend to exclude anti-inflammatory ω-3 PUFAs, and, more typically, contain high concentrations of proinflammatory ω-6 PUFAs, including linoleic acid (C18:2 ω-6; LA) and arachidonic acid (C20:4 ω-6; ARA) found in plant- and animal-derived lipids. Since Americans consume many times more ω-6s than ω-3s in the Western diet, their tissue phospholipid fatty acids skew heavily toward ω-3 insufficiency (19, 20). Several marine algae proficiently catalyze formation of DHA and EPA. Oily fish (e.g., salmon and mackerel) and small crustaceans (e.g., krill) bioconcentrate ω-3s into their membrane phospholipids by consuming marine algae (21). Individuals can increase DHA and EPA tissue incorporation, and correct ω-3 insufficiency, by consuming fish or dietary supplements with fish oil, krill oil, or microalgal oil. Intriguingly, ω-3 supplementation may be exploitable as a personalized medicine approach for individuals suffering from chronic inflammatory and autoimmune diseases to reduce dose and frequency of current therapies such as glucocorticoids that have myriad adverse effects.

Omega-3-rich fish oil supplementation has been shown to suppress autoantibody production, inflammatory gene expression, glomerulonephritis, and death from kidney failure in several different strains of lupus-prone mice (22–27), with DHA-enriched fish oil having the greatest potency (28, 29). Remarkably, we have found that dietary supplementation with DHA at realistic human equivalent Furthermore, we have demonstrated that pre-treating macrophages with DHA inhibited inflammasome activation by cSiO_2_ and linked this observation to suppression of NF-κB-driven proinflammatory genes (30). Understanding how DHA influences cSiO_2_-induced transcription signatures *in vivo* could provide insights into the underlying mechanisms by which ω-3s interfere with lupus flaring. In this investigation, we employed tissues from two recent published studies (17, 31) to test the hypothesis that DHA consumption interferes with upregulation of critical genes associated with cSiO_2_-triggered murine lupus. The results indicate that dietary DHA supplementation at clinically realistic levels impaired cSiO_2_-triggered expression of IFN- and chemokine-related genes that are likely to play critical roles in autoimmune pathogenesis and glomerulonephritis.

## Materials and Methods

### Animals and Diets

This investigation used materials and methods that have been more fully described in two previous published studies by our laboratory (17, 31). Experiments were approved by the Institutional Animal Care and Use Committee at Michigan State University (AUF #01/15-021-00). In both studies, female lupus-prone NZBWF1 mice (Jackson Laboratories, Bar Harbor, ME) were fed one of three diets that were based on the purified American Institute of Nutrition (AIN)-93G diet containing 70 g/kg fat (32). All diets contained 10 g/kg corn oil to ensure adequate basal essential fatty acids. The control diet (CON) contained 60 g/kg high-oleic safflower oil (Hain Pure Food, Boulder, CO). For DHA diets, high-oleic safflower oil was substituted with 10 g/kg (DHA low) or 25 g/kg (DHA high) microalgal oil containing 40% DHA (DHASCO, DSM Nutritional Products, Columbia MD). Resultant experimental diets contained 4 or 10 g/kg DHA, respectively, that equated, on a caloric basis, to human doses of 2 and 5 g per day, respectively. To prevent lipid oxidation, experimental diets were mixed weekly and stored at −20°C until use. Fresh feed was provided *ad libitum* to mice every 2 days.

### Experimental Design

**Figure 1:**
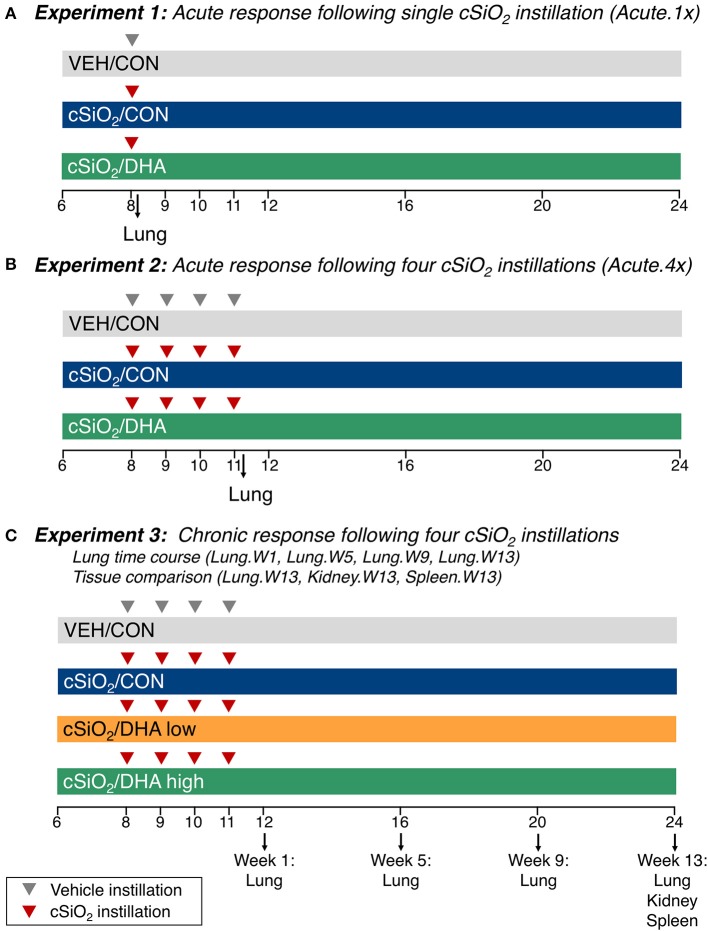
Design of experiments. At 8 weeks of age, female NZBWF1 mice were dosed intranasally with 25 μl PBS (VEH) or 25 μl PBS containing 1.0 mg cSiO_2_ once [experiment 1 **(A)**] or weekly for 4 weeks [experiments 2 and 3 **(B–C)**]. In experiments 1 and 2, mice were fed either a control diet (CON) or a diet supplemented with 5 g/kg DHA. In experiment 3, mice were fed either CON diet or diets supplemented with 2 g/kg DHA (low) or 5 g/kg DHA (high). Cohorts (*n* = 8) of mice were euthanized and necropsied 1 day (experiment 1 and 2) following the only/final instillation or 12, 16, 20, or 24 weeks of age corresponding to 1, 5, 9, or 13 weeks post the final instillation (experiment 3). Tissues obtained for nCounter digital transcript counting (NanoString PanCancer Immune Profiling gene set) are indicated above. In this manuscript, the primary comparisons of interest are the DHA-supplemented groups vs. the CON diet groups in cSiO_2_-exposed mice within each experiment. Please see Bates et al. (17) for detailed presentation and analysis of the impact of cSiO_2_ on gene expression vs. vehicle-exposed mice.

Experimental designs are depicted in Figure 1. For the acute studies (17), groups of 6 week old mice (*n* = 8) were fed CON or DHA high diets for the duration of the experiment. To model the acute response to one dose of cSiO_2_ (Acute.1x), a cohort of mice were anesthetized with 4% isoflurane and intranasally instilled with 1.0 mg cSiO_2_ (Min-U-Sil-5, 1.5–2.0 μm average particle size, Pennsylvania Sand Glass Corporation, Pittsburgh, PA) in 25 μl PBS or 25 μl PBS vehicle (VEH) (Figure 1A). To assess acute responses to short-term repeated exposure to cSiO_2_ (Acute.4x), a second cohort of mice received 1.0 mg cSiO_2_ or VEH once weekly for 4 weeks (Figure 1B). Cohorts were euthanized 24 h after the last cSiO_2_ instillation. Caudal lung lobes were removed, held in RNAlater (Thermo Fisher Scientific, Wilmington, DE) for 16 h at 4°C, and then stored at −80°C until RNA isolation. For the time course study (31), groups of mice were treated with VEH or cSiO_2_ weekly for 4 weeks beginning at age 8 weeks, (Figure 1C). Afterward, cohorts were terminated at 1, 5, 9, and 13 weeks post final cSiO_2_ exposure and organs collected and stored in RNAlater as described above. Lungs were analyzed at 1 (Lung.W1), 5 (Lung.W5), 9 (Lung.W9), and 13 (Lung.W13) weeks post cSiO_2_ exposure; spleens (Spleen.W13) and kidneys (Kidney.W13) were analyzed at 13 weeks. These times correspond with pathological changes previously reported in NZBWF1 mice after cSiO_2_ exposure preceding and through glomerulonephritis onset (15, 16, 31). Fatty acid concentrations in erythrocytes were analyzed by gas liquid chromatogorphy at OmegaQuant (Sioux Falls, SD).

### Gene Expression Analysis With NanoString nCounter

Total RNA was isolated from lung, spleen, and kidney using TriReagent (Sigma Aldrich, St. Louis, MO) and RNeasy Mini Kits with DNase treatment (Qiagen, Valencia, CA). RNA integrity (RIN values > 7.0) in samples was verified using an LabChip Gx Analyzer (Caliper Life Sciences, Waltham, MA). RNA (*n* = 7–8/group) was analyzed utilizing the nCounter Mouse PanCancer Immune Profiling Panel (catalog # 115000142, probe annotations available in Supplementary File 1) as described in detail previously (17) (Supplementary Figures 1–3). NanoString's software nSolver v3.0.22 was utilized for differential gene expression analyses as outlined previously (17) and depicted in Supplementary Figure 4. Statistically significant, differentially expressed genes were delineated as those with expression levels corresponding to a 1.5-fold change with respect to the corresponding CON diet group and a false discovery rate (Benjamini–Hochberg method) *q* < 0.05 (Supplementary Figure 5). nSolver differential expression analysis outputs from are contained in Supplementary File 2. BioVenn (33) or Venny v2.1 (34) was used to produce Venn diagrams of significant differentially expressed genes in cSiO_2_ groups.

Annotated gene sets, global, and directed significance scores were calculated for each pathway to ascertain the effects of treatments as previously described (17). Global scores estimate the cumulative evidence for the differential expression of genes for specific pathway, whereas directed significance scores reflect tendency for pathway genes to be over- or under-expressed collectively. Additionally, pathway Z scores were used to summarize data from a pathway's genes into a single score calculated as the first principal component of the pathway genes' normalized expression and standardized by Z scaling. ClustVis (35) was employed to carry out unsupervised hierarchical cluster analyses (HCC) and principal components analyses (PCA) using log_2_ transcript count data. Summary tables for all significance and pathway Z scores can be found in Supplementary Files 3, 4.

Spearman rank correlations were done to assess overall patterns in the gene expression profiles compared to percent CD45R+ (B cells) and CD3+ (T cells) in lung tissues as markers for ectopic lymphoid tissue development (31) and with the percent of ω-3 highly unsaturated fatty acids (HUFA; fatty acids with 20 or more carbons and three or more double bonds) in the total HUFA of erythrocytes (ω-3 HUFA score) (19). Correlation analysis was conducted using *cor* and *corrplot* functions in R (www.R-project.org). Spearman ρ values were determined utilizing individual sample pathway Z scores and phenotype data from mice from 1, 5, 9, or 13 weeks cohorts (31). A correlation was considered significant when ρ > 0.5 or <-0.5 and *p* < 0.05.

STRING database version 10.5 (http://string-db.org/) was used for network analyses for interactions among significant genes significant genes identified by the nSolver data analysis at a confidence level for associations set at ≥0.7. Clusters were identified using the Markov Cluster (MCL) algorithm with inflation parameter of 1.5. Networks produced by STRING were mapped with Cytoscape v3.0, with nodes indicating significant genes and edge width designating combined interaction score. Data for STRING-db networks and the predicted clusters, including protein-protein interactions and functional annotations can be found in Supplementary File 5.

### Immunofluorescence Microscopy

Mouse lungs (*n* = 2 to 3 per group) were fixed in 4% paraformaldehyde, embedded in paraffin, and cut into 5 μm thick sections by the histology core at Michigan State University. The lung tissue sections were then deparaffinized by incubation for 1 h at 60°C, followed by immersion in xylene for 15 min with two changes. Tissues were rehydrated by sequential 10 min incubations in 100, 90, 70, and 50% (v/v) ethanol, followed by two 5 min incubations with deionized water. Epitope retrieval was accomplished by 10 min incubation in 10 mM sodium citrate buffer (pH 6.0), followed by another 5 min wash in deionized water. Tissues were permeabilized by incubation for 15 min in 1% (v/v) goat serum containing 0.4% (v/v) Triton X-100 in PBS (PBST). Blocking of non-specific binding was done by incubation in 5% (v/v) goat serum in PBST for 30 min at room temperature. Detection of Mx1 and Oas2 proteins was accomplished by incubation with primary polyclonal antibodies (Mx1 catalog no. 1370-1-AP and Oas2 catalog no. 1927-1-AP; Proteintech, Rosemont, IL) diluted to 1:50 in 1% goat serum PBST and incubation overnight at 4°C in a humidified chamber. Next, tissue sections were washed twice with 1% goat serum PBST for 10 min and then incubated with Alexa Fluor^TM^ 594 goat anti-rabbit secondary antibody (Invitrogen, Carlsbad, CA) diluted to 1:1000 in 1% goat serum PBST at room temperature for 1–2 h in the dark. Sections were rinsed twice with PBST for 10 min, and the nuclei were counterstained by incubating overnight in Prolong^TM^ gold antifade reagent with DAPI (Invitrogen). Samples were stored in the dark until imaged using the Evos FL Auto 2 cell imaging system; 5 to 6 fields of view for each animal for each treatment group were inspected qualitatively.

### Enzyme-Linked Immunosorbent Assay for Cxcl10

The concentration of Cxcl10 protein in whole lung homogenate was determined by ELISA using a the mouse Cxcl10 DuoSet kit (R&D Systems, Minneapolis, MN) according to the manufacturer's instructions. Briefly, snap-frozen lungs were thawed, weighed, and homogenized in cold lysis buffer containing protease inhibitors. Homogenates were then centrifuged at 15,000 × *g* for 20 min at 4°C, and the supernatants were used for measuring Cxcl10 by ELISA. Total protein concentrations in the lung tissue homogenates were determined using the Pierce BCA protein assay kit (ThermoFisher, Waltham, MA).

## Results

**Figure 2:**
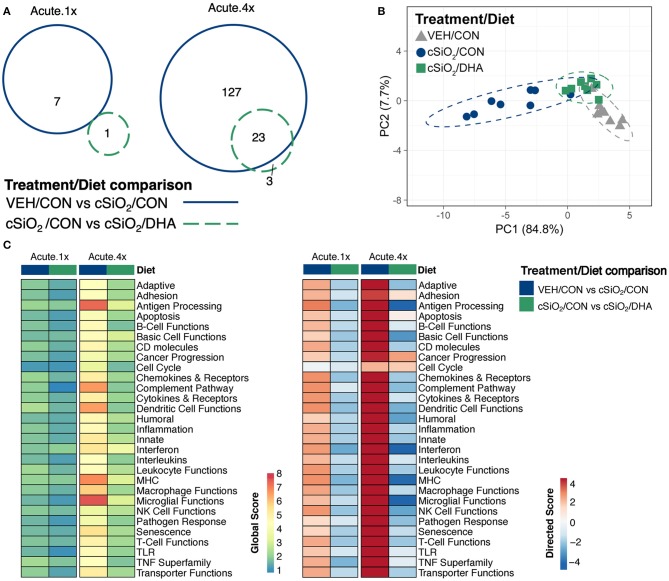
Acute transcriptional response of immune-associated genes in DHA-supplemented mice that received either a single or four weekly instillations of cSiO_2_. **(A)** Venn diagram depicting overlap of genes differentially regulated by exposure to cSiO_2_ compared to those differentially regulated by DHA supplementation (FDR *q* < 0.05, 1.5-fold change). The overlap region indicates genes affected by cSiO_2_ exposure that were also altered by DHA supplementation. **(B)** PCA plot of differentially expressed genes for mice in the Acute.4x dosing group compared to a dosing-matched vehicle control group fed CON diet (VEH/CON). PC1 and PC2 are shown with 95% confidence interval bands (dashed ellipses). A PCA plot is not shown for the Acute.1x dosing group as only one gene was identified as differentially regulated by DHA for that dosing protocol. Hierarchical cluster analyses are provided in Supplementary Figure 6. **(C)** Global and directed significance scores for immune pathways were determined using nSolver (see section Materials and Methods) by comparing mice in the cSiO_2_/CON group to dosing-matched vehicle (VEH) controls fed CON diet or by comparing mice in the cSiO_2_/DHA group vs. cSiO_2_-exposed, CON-fed mice.

Acute immune gene responses 1 day after single (Acute.1x) or repeated (Acute.4x) intranasal dosing with cSiO_2_ were compared in mice fed CON or DHA high diets (Figures 1A,B). Transcriptomic analyses revealed that that 7 and 140 genes were differentially regulated (FDR *q* < 0.05, 1.5-fold change) in the lung 1 day after cSiO_2_ treatment in the Acute.1x and Acute.4x groups, respectively (Figure 2A). While DHA consumption did not affect cSiO_2_-induced changes in the single dose group, 23 genes were affected by DHA in mice treated with multiple doses of the particle. Principal component analysis of the Acute.4x responses indicated that DHA-fed cSiO_2_-treated mice clustered closely with the CON-fed VEH-treated mice, with both clusters being relatively distinct from CON-fed cSiO_2_-treated mice (Figure 2B). Heat mapping of global and directed significance scores showed that cSiO_2_-potentiated pathways were largely attenuated by DHA consumption (Figure 2C).

**Figure 3:**
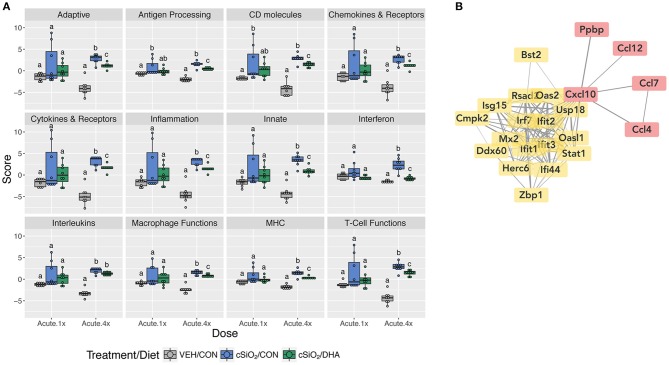
Pathway Z scores and network visualization for acute transcriptional response of immune-associated genes in DHA-supplemented mice that received either a single or four weekly instillations of cSiO_2_. **(A)** For cSiO_2_-exposed mice, gene expression pathway scores were calculated as the first principal component of the pathway genes' normalized expression and standardized by Z scaling. Immune pathway Z scores are presented as Tukey box-plots (*n* = 8) for select immune pathways of interest. Different letters indicate treatment/diet groups are significantly different (*p* < 0.05) as determined by the Steel-Dwass nonparametric test for all pairs. Heatmaps depicting individual pathway Z scores for all pathways captured by the NanoString PanCancer Immune Profiling gene panel are provided in Supplementary Figure 7. **(B)** Network interactions were modeled using the STRING database (string-db.org) with a minimum required interaction score ≥0.7, and clusters were identified using the Markov Cluster (MCL) algorithm with inflation parameter of 1.5. The network was visualized in Cytoscape, and edge widths reflect the combined interaction score (thicker edges indicate higher score). Note, a network for mice treated only once with cSiO_2_ (Acute.x1) was not made, as only one gene was significantly affected by DHA supplementation.

When gene expression pathway scores were calculated as the first principal component of the pathway genes' normalized expression and standardized by Z scaling, several cSiO_2_-induced immune pathways were found to be significantly downregulated by DHA supplementation in the Acute.4x group (Figure 3A; Supplementary Figure 7). Affected genes included those associated with inflammation; innate and adaptive immunity; IFN, chemokines, interleukins, cytokines; T-cell and macrophage function; and antigen processing and MHC expression. Network mapping showed that both IFN- and chemokine-related pathways were among the most prominently affected by DHA (Figure 3B).

**Figure 4:**
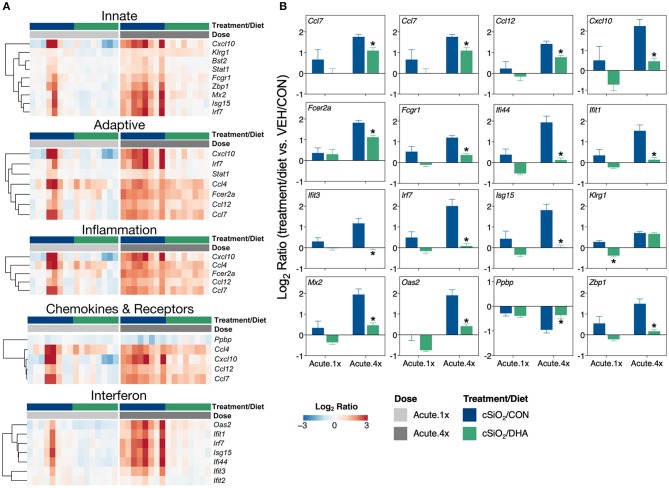
Comparison of DHA-responsive genes involved in immune response in lung tissues of mice that received either a single or four weekly instillations of cSiO_2_. Gene expression data are shown as log_2_ ratios for cSiO_2_-exposed mice fed either CON or DHA-supplement diets calculated with respect to dosing-matched, vehicle-exposed, CON-fed mice (VEH/CON, log_2_ ratio = 0). **(A)** For the selected immune pathways shown, heatmaps with unsupervised clustering (Euclidian distance method) by gene depict log_2_ expression values for all genes identified as significantly differentially expressed (FDR *q* < 0.05, 1.5-fold change) after a single (Acute.1x) or four repeated weekly doses (Acute.4x) of cSiO_2_. **(B)** The mean log_2_ ratio values + SEM for selected genes of interest are also shown. *, *p*<0.05 for DHA compared to CON diet as determined by nSolver statistical analyses (see Supplementary File 2 for test specifications and FDR-corrected *q* values).

DHA's effects on representative pathway genes are depicted as heat maps and line plots in Figure 4. While only a few of the eight mice in the Acute.1x group responded strongly to cSiO_2_, the responses were very similar to those seen in all eight cSiO_2_-treated mice in the Acute.4x group (Figure 4A). DHA supplementation affected all cSiO_2_-induced genes by downregulation (Supplementary Figure 6). Consistent with the network analysis (Figure 3B), DHA significantly suppressed the upregulation of the IFN-related genes *Zbp1, Mx2, Oas2, Ifit1, Ifit3, Ifit3, Irf7, Isg15*, and *Ifi44* and the chemokine-associated genes *Ccl4, Cxcl10, Ccl7, Ccl12* (Figure 4B).

**Figure 5:**
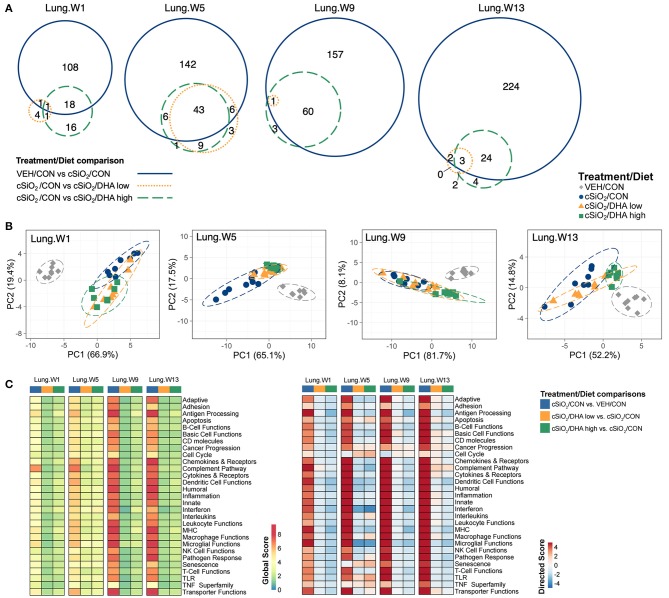
Effect of DHA supplementation on cSiO_2_-induced transcriptional changes in lung tissues of mice 1, 5, 9, or 13 weeks post instillation. **(A)** Venn diagrams depicting overlap of genes differentially regulated by exposure to cSiO_2_ compared to those differentially regulated by supplementation with DHA low or DHA high diets (FDR *q* < 0.05, 1.5-fold change). The overlap regions indicate genes affected by cSiO_2_ exposure that were also altered by DHA supplementation. Hierarchical cluster analyses are provided in Supplementary Figure 9. **(B)** Principal components analyses of differentially expressed genes in lung tissues of DHA-supplemented mice exposed to cSiO_2_ at 1, 5, 9, or 13 weeks post instillation compared to time-matched vehicle (VEH/CON) and cSiO_2_-exposed (cSiO_2_/CON) control diets. PC1 and PC2 are shown with 95% confidence interval bands (dashed ellipses). **(C)** Global and directed significance scores for immune pathways were determined using nSolver (see section Materials and Methods) by comparing mice in the cSiO_2_/CON group to time-matched vehicle (VEH) controls fed CON diet or by comparing mice in the cSiO_2_/DHA low or the cSiO_2_/DHA high group vs. cSiO2-exposed, CON-fed mice.

The effect of DHA low and high diets on chronic mRNA responses to short-term repeated cSiO_2_ were assessed in the lung over a 13 week period (Figure 1C). cSiO_2_ exposure elicited differential expression (FDR *q* < 0.05, 1.5-fold change) in the lung of 128, 197 genes, 218, and 253 genes at 1, 5, 9, and 13 weeks PI, respectively (Figure 5A). DHA low diet influenced 2, 49, 1, and 5 genes at these timepoints, respectively, whereas, the DHA high diet, affected 19, 49, 61, and 27 genes, respectively. Principal component analysis indicated strong separation of VEH-treated mice fed CON diet from all cSiO_2_-treated mice at all time points (Figure 5B). cSiO_2_-treated DHA low-fed mice responses clustered closely with cSiO_2_-treated DHA high-fed mice at 1 and 5 weeks PI, and with cSiO_2_-treated CON-fed mice at 9 and 13 weeks PI. Finally, cSiO_2_-treated DHA high-fed mice clustered distinctly from cSiO_2_-treated CON-fed mice at all time points. Hierarchal cluster analysis indicated that most of these genes were upregulated by cSiO_2_ treatment and suppressed by DHA (Supplementary Figure 8).

**Figure 6:**
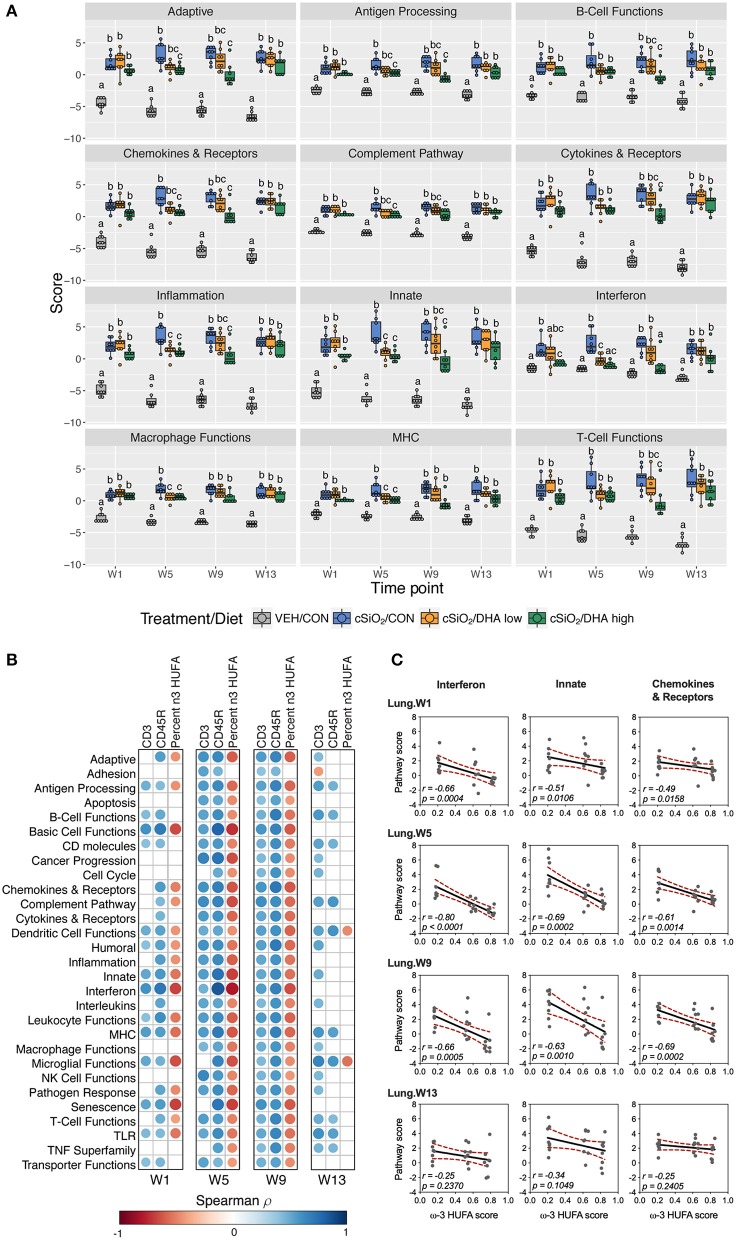
Lung tissue pathway Z scores and correlation analyses of immune-associated pathways. **(A)** Pathway Z scores are presented as Tukey box-plots (*n* = 8) for select immune pathways of interest. Different letters indicate treatment/diet groups are significantly different (*p* < 0.05) as determined by the Steel-Dwass non-parametric test for all pairs. Heatmaps depicting individual pathway Z scores for all pathways captured by the NanoString PanCancer Immune Profiling gene panel are provided in Supplementary Figure 9. **(B)** For all cSiO_2_-treated groups, spearman ρ values were calculated by correlating pathway Z scores with percent positive staining tissue (CD3 and CD45R) or the percent ω-3 HUFA in erythrocytes (ω-3 HUFA score). Significant correlation values (*p*<0.05) are represented as circles colored by the correlation value (blue, positive; red, negative); non-significant correlations are indicated by blank cells. **(C)** Scatter plots for pathway scores vs. the diet ω-3 HUFA score for selected pathways of interest. Linear regression lines with 95% confidence intervals (dashed red line) are shown along with the Spearman *r* value and *p-*value.

As observed in the Acute.4x study, DHA affected chronic expression of genes altered by cSiO_2_ exposure related to inflammation; innate and adaptive immunity; IFN, chemokines cytokines; B-cell, T-cell, and macrophage function; MHC expression and antigen processing; and complement (Figures 5C, 6A; Supplementary Figure 9). Most pathways in individual lungs of cSiO_2_-exposed lupus-prone mice time-dependently correlated with the presence of B cells and T cells (markers of ectopic lymphoid neogenesis) in the same lung tissues reported in the parent study (31) (Figures 6B,C). Significantly, most of these gene pathways were negatively correlated with ω-3 HUFA scores in erythrocytes from corresponding animals, with the strongest response noted for the IFN pathway at week 5.

**Figure 7:**
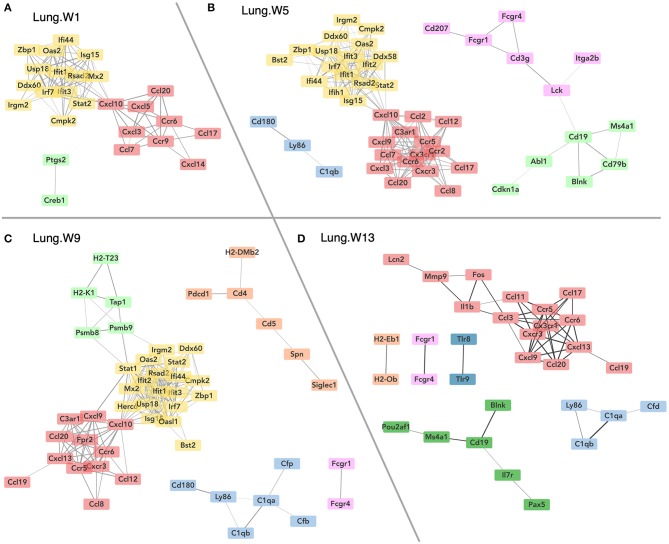
Network visualization of genes significantly affected by DHA supplementation in lung tissues obtained 1 **(A)**, 5 **(B)**, 9 **(C)**, or 13 **(D)** weeks post instillation with cSiO_2_. Network interactions for genes differentially regulated by either DHA low or DHA high supplementation at each time point were modeled using the STRING database (string-db.org) with a minimum required interaction score ≥0.7, and clusters were identified using the Markov Cluster (MCL) algorithm with inflation parameter of 1.5. The network was visualized in Cytoscape, and edge widths reflect the combined interaction score (thicker edges indicate higher score).

Figure 7 illustrates gene networks affected by dietary DHA supplementation during the course of cSiO_2_-induced disease development in the lungs. Consistent with the Acute.4x findings, DHA dramatically affected IFN- and chemokine-related genes at 1, 5, 9 weeks PI and, to a lesser extent, at 13 weeks PI. Also of note, expression of genes associated with the complement pathway (*C1qb, C1qa, Cfd*, and *Cfb*) was affected at weeks 5, 9, and 13 PI and with B-cell signaling and differentiation (*Pou2af1, Ms4a1, Cd19, Pax5*, and *Blnk*) at week 13 PI.

**Figure 8:**
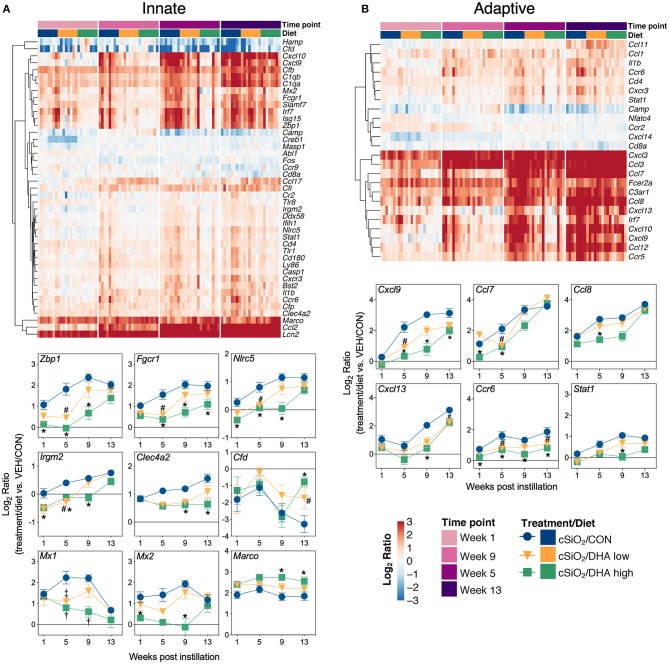
Time course of DHA-responsive genes associated with innate and adaptive immune pathways in lung tissues of mice 1, 5, 9, or 13 weeks post instillation with cSiO_2_. Gene expression data were obtained using the NanoString PanCancer Immune Profiling gene panel and are shown as log_2_ ratios for cSiO_2_-exposed mice fed CON, DHA low, or DHA high diets calculated with respect to time-matched, vehicle-exposed, CON-fed controls (VEH/CON; log_2_ ratio = 0). For innate **(A)** or adaptive **(B)** immune pathways, heatmaps with unsupervised clustering (Euclidian distance method) by gene depict log_2_ expression values for all genes identified as significantly differentially expressed (FDR *q* < 0.05, 1.5-fold change) at any one of the indicated time points. The mean log_2_ ratio values ± SEM for selected genes of interest are also shown. *p < 0.05 for DHA high compared to CON diet and ^#^*p* < 0.05 for DHA low compared to CON diet as determined by nSolver statistical analyses (see Supplementary File 2 for test specifications and FDR-corrected *q-*values). †*p* < 0.05 for DHA high compared to CON diet and ‡*p* < 0.05 for DHA low compared to CON diet as determined by non-parametric Kruskal–Wallis test (for *Mx1* only).

**Figure 9:**
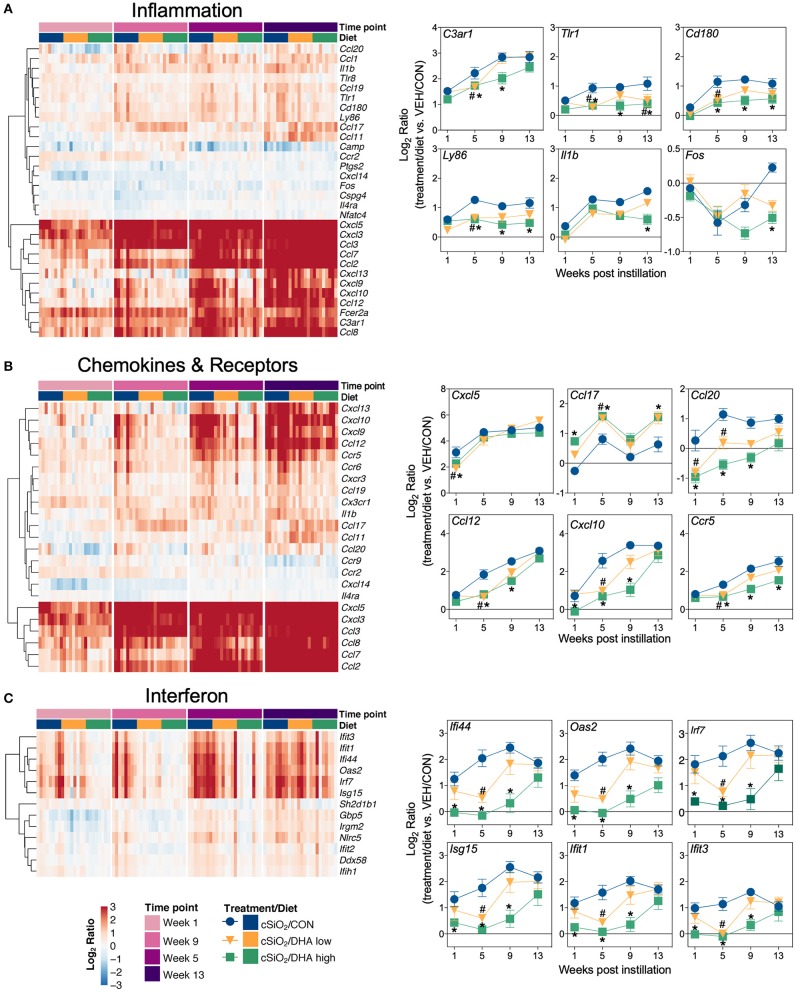
Time course of DHA-responsive genes associated with inflammation, chemokines & receptors, or immune pathways in lung tissues of mice 1, 5, 9, or 13 weeks post instillation with cSiO_2_. Gene expression data were obtained using the NanoString PanCancer Immune Profiling gene panel and are shown as log_2_ ratios for cSiO_2_-exposed mice fed CON, DHA low or DHA high diets calculated with respect to time-matched, vehicle-exposed, CON-fed controls (VEH/CON; log_2_ ratio = 0). For inflammation **(A)**, chemokines and receptors **(B)**, or interferon **(C)** pathways, heatmaps with unsupervised clustering (Euclidian distance method) by gene depict log_2_ expression values for all genes identified as significantly differentially expressed (FDR *q* < 0.05, 1.5-fold change) at any one of the indicated time points. The mean log_2_ ratio values ± SEM for selected genes of interest are also shown. *p < 0.05 for DHA high compared to CON diet and ^#^*p* < 0.05 for DHA low compared to CON diet as determined by nSolver statistical analyses (see Supplementary File 2 for test specifications and FDR-corrected *q* values).

Heat maps and line plots as a function of time were constructed for representative genes associated with innate and adaptive immunity (Figures 8A,B) and with inflammation, IFN, and chemokines (Figures 9A–C). Particularly striking was the impact of DHA on IFN and chemokine genes, which were among the earliest and most highly suppressed. Specifically, consumption of the DHA low diet significantly suppressed cSiO_2_-induced gene expression at 1 week post installation (PI) and/or 5 weeks PI (e.g., *Ccl12, Ccl20, Cxcl10, Oas2, Isg15*, and *Ifit1*), whereas effects of the DHA high diet were longer lasting with significant effects also being observed at 9 weeks PI (*Mx2, Cxcl10, Ccl12, Ifi44, Oas2, Ift1*) and 13 weeks PI (e.g., *Il1b, Fgcr1, Cxcl9*).

**Figure 10:**
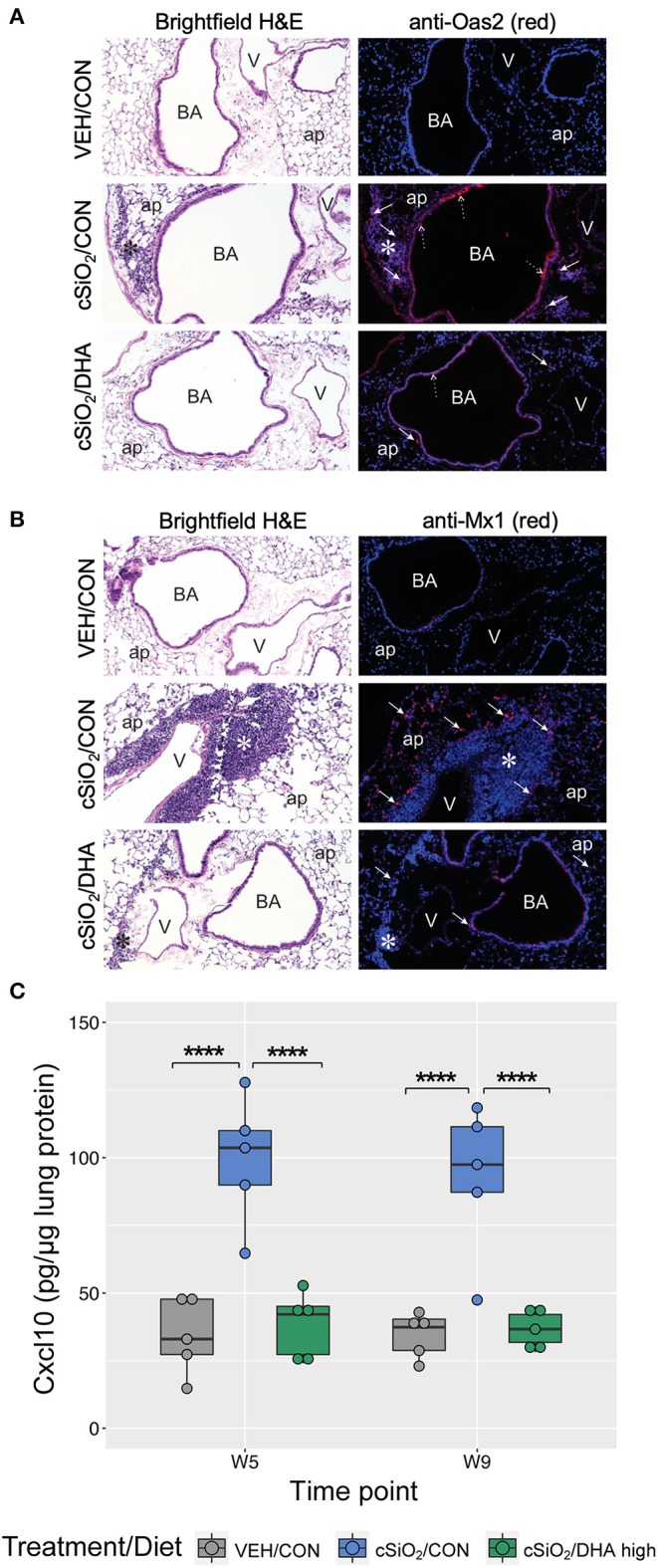
Immunofluorescence detection of interferon-responsive genes Oas2 and Mx1 in lung tissues at 9 weeks post instillation with cSiO_2_ and expression of Cxcl10 in lung homogenates. **(A,B)** Representative light photomicrographs depict H&E-stained lung sections from VEH/CON, cSiO_2_/CON, and cSiO_2_/DHA high groups at 9 wk post-installation, while representative fluorescence microscopy images depict immunofluorescence staining of the same tissues for either Oas2 **(A)** or Mx1 **(B)** proteins (red channel) and Hoechst stain for nuclei (blue channel). For **(A)**, Oas2-expressing cells are apparent in the ectopic lymphoid tissue (solid arrow) and the airway epithelium (dashed arrow). For **(B)**, Mx1-expressing cells are apparent in the alveolar parenchyma (solid arrow). ap, alveolar parenchyma; BA, bronchiolar airway; V, blood vessel, *, ectopic lymphoid tissues; solid arrow, MX1-positive staining cells in the alveolar parenchyma. **(C)** Expression values for Cxcl10 protein in lung homogenates are shown as Tukey box-plots (*n* = 5). ****p < 0.0001 for comparisons among treatment groups within each time point as determined by two-way ANOVA (main effect of time point *p* = 0.6785; main effect of treatment group *p* < 0.0001; interaction *p* = 0.8908).

Immunofluorescence microscopy of lung tissues of mice obtained a 9 wk PI with cSiO_2_ revealed increased expression of Oas2 protein in ectopic lymphoid tissues and the airway epithelium, whereas dietary supplementation with DHA appeared to suppress expression of Oas2 at these sites (Figure 10A). Similarly, DHA supplementation suppressed the over-expression of Mx1 protein in the alveolar parenchyma triggered by cSiO_2_ exposure (Figure 10B). Of note, while *Mx1* gene expression was induced by cSiO_2_ and then repressed by DHA, these changes in gene expression were not statistically significant as determined by the nSolver data analysis workflow. This result was likely due to failure of the mean to meet the threshold (10× background signal) for some treatment groups resulting in the use of the much less powerful Wald test. Separate analysis using the Kruskal-Wallis non-parametric test (GraphPad Prism, San Diego, CA) suggested that DHA supplementation indeed suppressed *Mx1* expression induced by silica treatment at 5 and 9 weeks post installation (Figure 8A), a determination that agrees with the immunofluorescence microscopy results (Figure 10B). Lastly, measurement of Cxcl10 protein (also known as interferon gamma protein 10 (IP-10) or small-inducible cytokine B10) in lung homogenate using a standard ELISA revealed a profound 3-fold increase in its expression in tissues of cSiO2-exposed mice at both 5 and 9 weeks PI (Figure 10C). Remarkably, dietary supplementation with DHA entirely blocked that response such at Cxcl10 expression was not different from VEH/CON mice.

**Figure 11:**
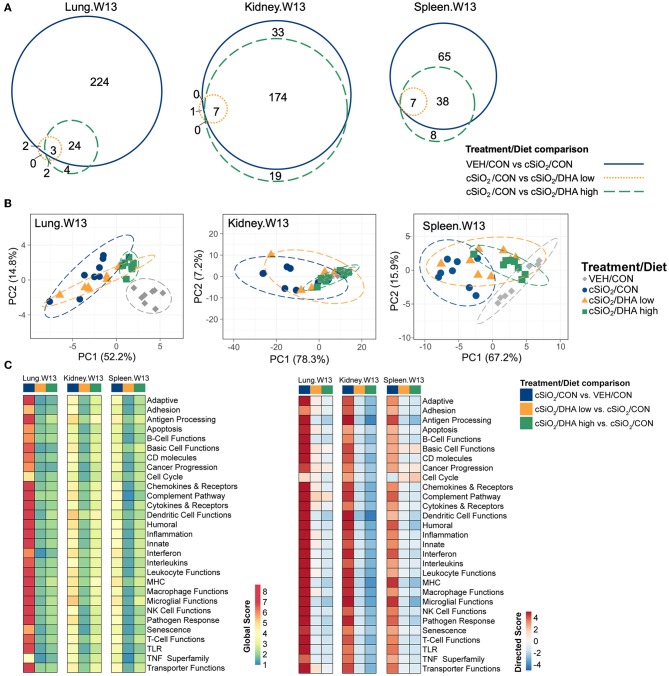
Effect of DHA supplementation on cSiO_2_-induced transcriptional changes in lung, kidney or spleen tissues of mice 13 weeks post instillation. **(A)** Venn diagrams depicting overlap of genes differentially regulated by exposure to cSiO_2_ compared to those differentially regulated by supplementation with DHA low or DHA high diets (FDR *q* < 0.05, 1.5-fold change). The overlap regions indicate genes affected by cSiO_2_ exposure that were also altered by DHA supplementation. Hierarchical cluster analyses are provided in Supplementary Figure 10. **(B)** Principal components analyses of differentially expressed genes compared to tissue-matched vehicle control (VEH/CON) and cSiO_2_-exposed (cSiO_2_/CON) control diets. PC1 and PC2 are shown with 95% confidence interval bands (dashed ellipses). **(C)** Global and directed significance scores for immune pathways were determined using nSolver (see section Materials and Methods) by comparing mice in the cSiO_2_/CON group to dosing-matched vehicle (VEH) controls fed CON diet or by comparing mice in the cSiO_2_/DHA low or the cSiO_2_/DHA high group vs. cSiO2-exposed, CON-fed mice.

The effects of DHA supplementation on cSiO_2_-induced transcriptional changes were compared in lung, kidney and spleen tissues of mice at 13 weeks PI (Figure 11A; Supplementary Figure 10). Consumption of the DHA high diet influenced 11 percent of cSiO_2_-affected genes in the lung at this timepoint, while in the kidney and spleen, 85 and 59 percent of the induced transcriptomes were modulated, respectively. Many fewer cSiO_2_-altered genes in the lung (1%), kidney (3%), and spleen (5%) were affected in the mice fed the DHA low diet. Principal component analyses of the kidney indicated close associations among VEH/CON, cSiO_2_/DHA low, and cSiO_2_/DHA high groups as compared to cSiO_2_/CON group (Figure 11B). In the spleen, there were substantial overlaps between the VEH/CON and cSiO_2_/DHA high groups and between the cSiO_2_/DHA low and cSiO_2_/CON groups.

**Figure 12:**
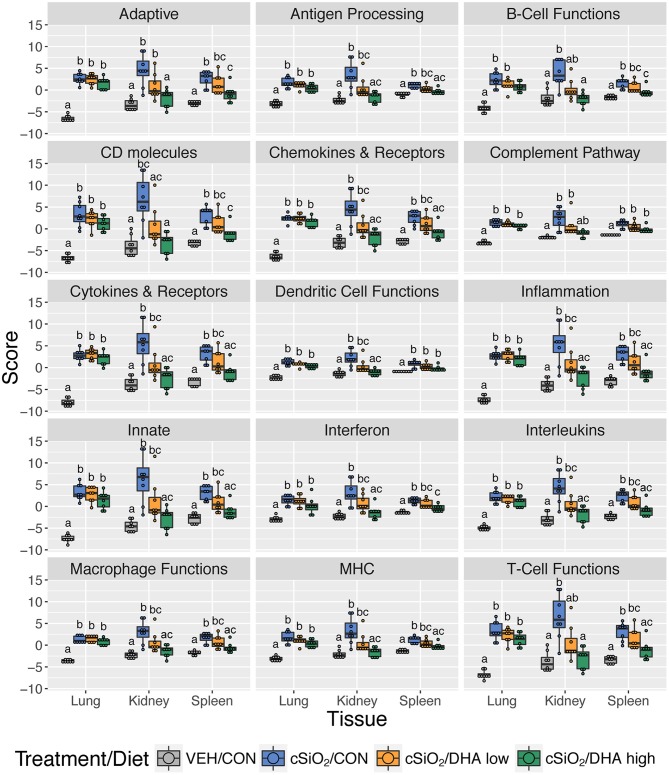
Effect of DHA supplementation on selected immune pathways in lung, kidney and spleen tissues of mice 13 weeks post instillation. Mice fed either CON, DHA low, or DHA high diets received four repeated weekly doses of cSiO_2_ via intranasal instillation. Gene expression was determined by nCounter digital transcript counting in lung, kidney or spleen tissues obtained 13 weeks post instillation. Pathway Z scores are presented as Tukey box-plots (*n* = 8) for select immune pathways of interest. Different letters indicate treatment/diet groups are significantly different (*p* < 0.05) as determined by the Steel-Dwass non-parametric test for all pairs. Heatmaps depicting individual pathway Z scores for all pathways captured by the NanoString PanCancer Immune Profiling gene panel are provided in Supplementary Figure 11.

**Figure 13:**
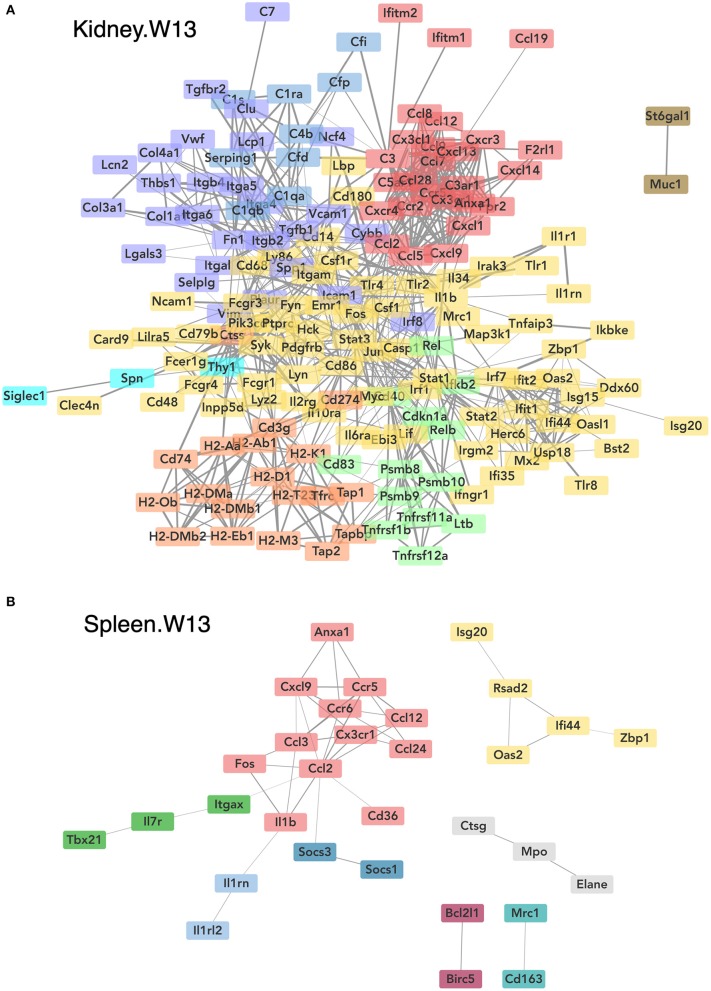
Network visualization of genes significantly affected by DHA supplementation in kidney **(A)** or spleen **(B)** tissues obtained 13 weeks post instillation with cSiO_2_. Network interactions for genes differentially regulated by either DHA low or DHA high supplementation at each time point were modeled using the STRING database (string-db.org) with a minimum required interaction score ≥0.7, and clusters were identified using the Markov Cluster (MCL) algorithm with inflation parameter of 1.5. The network was visualized in Cytoscape, and edge widths reflect the combined interaction score (thicker edges indicate higher score).

**Figure 14:**
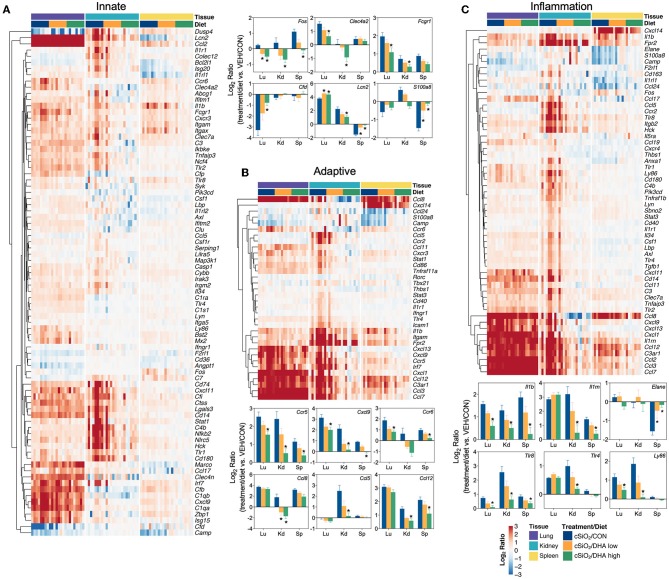
Comparison of DHA-responsive genes associated with innate, adaptive, and inflammation immune pathways in lung, kidney, or spleen tissues 13 weeks post instillation with cSiO_2_. Gene expression data were obtained using the NanoString PanCancer Immune Profiling gene panel and are shown as log_2_ ratios for cSiO_2_-exposed mice fed CON, DHA low or DHA high diets calculated with respect to tissue-matched, vehicle-exposed, CON-fed controls (VEH/CON; log_2_ ratio = 0). For the innate **(A)**, adaptive **(B)**, and inflammation **(C)** pathways, heatmaps with unsupervised clustering (Euclidian distance method) by gene depict log_2_ expression values for all genes identified as significantly differentially expressed (FDR *q* < 0.05, 1.5-fold change) in any one of the indicated tissues. The mean log_2_ ratio values + SEM for selected genes of interest are also shown. *p < 0.05 for DHA high compared to CON diet (see Supplementary File 2 for test specifications and FDR-corrected *q-*values).

**Figure 15:**
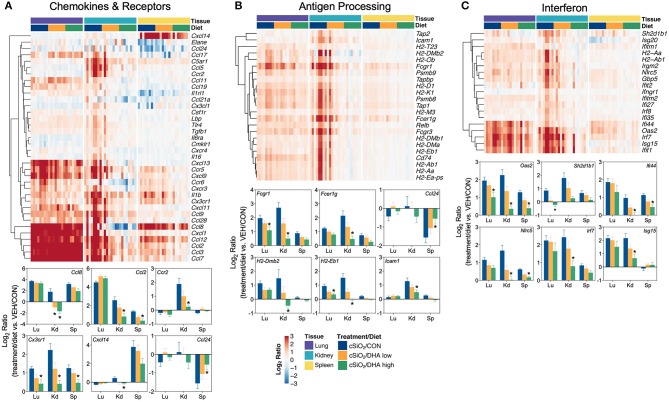
Comparison of DHA-responsive genes associated with chemokines & receptors, antigen processing and interferon immune pathways in lung, kidney, or spleen tissues 13 weeks post instillation with cSiO_2_. Gene expression data were obtained using the NanoString PanCancer Immune Profiling gene panel and are shown as log_2_ ratios for cSiO_2_-exposed mice fed CON, DHA low, or DHA high diets calculated with respect to tissue-matched, vehicle-exposed, CON-fed controls (VEH/CON; log_2_ ratio = 0). For the chemokines & receptors **(A)**, antigen processing **(B)**, and interferon **(C)** pathways, heatmaps with unsupervised clustering (Euclidian distance method) by gene depict log_2_ expression values for all genes identified as significantly differentially expressed (FDR *q* <0 .05, 1.5-fold change) in any one of the indicated tissues. The mean log_2_ ratio values + SEM for selected genes of interest are also shown. *p < 0.05 for DHA high compared to CON diet (see Supplementary File 2 for test specifications and FDR-corrected *q-*values).

Consistent with DHA's effects in the lung in earlier weeks, its supplementation affected a broad array of cSiO_2_-induced pathways in the kidney and spleen at week 13 (Figures 11C, 12, 13). Network analysis revealed that DHA had robust effects on critical genes associated with glomerulonephritis including those related to IFN signaling (e.g., *Irf7, Ifit1, Oas2, Isg15*); cytokines and chemokines (e.g., *Ccl8, Ccl2, Ccr2, Cx3cr1*); and antigen processing and MHC (e.g., *H2-Dmb2, Fcgr1, Fcer1g, H2-Eb1*) (Figure 13A). Lastly, heat mapping and line plotting revealed that DHA dose-dependently suppressed induction of many genes in the kidney associated with innate and adaptive immunity and inflammation (Figure 14), and chemokines, IFN and antigen processing (Figure 15), whereas the effects were much more modest in the spleen with only a few genes uniquely affected by DHA in this tissue (e.g., *Elane* and *Ccl124*).

## Discussion

DHA and other ω-3s potentially quell lupus flaring and progression by altering intracellular signaling, transcription factor activity, gene expression, bioactive lipid mediator production, and membrane structure and function [reviewed in (36)]. We show here for the first time how DHA supplementation at translationally relevant doses influenced cSiO_2_-induced changes in gene regulation in the NZBWF1 female mouse model. Over the course of the chronic study, DHA suppressed a broad array of cSiO_2_-induced inflammatory, innate, and adaptive gene responses in the lung that correlated with inhibition of ectopic lymphoid neogenesis previously described in these same tissues (31). Based on ELISA data in previous studies (15, 16), we expected proinflammatory genes to be critically affected here, however, cSiO_2_-induced genes specifically associated with the IFN signature and chemokines were among the earliest and most robustly downregulated by DHA treatments. Furthermore, we determined that expression of the IFN-responsive proteins Mx1, Oas2 and Cxcl10 in the lung was similarly markedly induced by cSiO_2_ treatment and were suppressed by dietary intervention with DHA. In the kidney, DHA suppressed the expression of a broad array of gene pathways related to inflammation, innate/adaptive immunity, IFN, chemokines, antigen processing that likely contribute to cSiO_2_-triggered glomerulonephritis. Finally, the observation that lupus-associated mRNA signatures negatively correlated with erythrocyte ω-3 HUFA scores is of high relevance from a translational perspective.

Investigation of cSiO_2_-triggered lupus in the NZBWF1 mouse offers an exquisite window for exploring how environmental factors contribute to this devastating autoimmune disease as well as for understanding how potential interventions might prevent or diminish SLE flaring and progression. At the mechanistic level, polymorphonuclear leukocytes (PMNs) and alveolar macrophages (AMΦs) are the primary responders to cSiO_2_ and other particles in the lung. Both cell types were increased in the alveolar fluids from the lungs of cSiO_2_-exposed NZBWF1 mice used for the present study (31). AMΦ death occurs following lysosomal membrane permeabilization with inflammasome activation and involves pyroptosis, apoptosis, and necrosis (37, 38). cSiO_2_ induces death in PMN by necroptosis, a process associated with release of neutrophil extracellular traps (NETs) (39, 40). Because cSiO_2_ clearance in animal models is limited (41–43), exposure to this particle drives a vicious cycle in AMΦs and PMNs involving phagocytosis of SiO_2_, cell death, autoantigen release, cSiO_2_ particle escape, and renewed cSiO_2_ phagocytosis [reviewed in (30)]. This feedback loop perpetuates recurrent pulmonary exposure to cSiO_2_ potentially saturating the efferocytotic capacity of the lung with cell corpses and autoantigens that can override tolerogenic mechanisms, particularly in animals genetically prone to autoimmunity, such as NZBWF1 mice (16, 31). In agreement with this scenario, in this study, cSiO_2_ induced expression of genes in the lung indicative of sustained IFN activity, chemokine release, cytokine production, complement activation, and adhesion molecule expression. These transcriptome signatures correlated with the particle's capability to evoke in the lung an early and persistent sterile inflammation, ectopic lymphoid tissue development, autoantibody production, and, in the longer term, elicit systemic autoimmunity and glomerulonephritis (17).

In the present study, short-term repeated exposure to cSiO_2_ evoked mRNA signatures in the lung that reflected wide-scale activation of inflammatory, innate, and adaptive gene pathways. Although comparable genes were elevated at 1 d and 1, 5, 9, and 13 weeks PI, the responses increased in both extent and intensity with time. This observation suggested that the effects of cSiO_2_ were not self-limiting and were consistent with a perpetual feedback loop. These gene pathways correlated with ectopic lymphoid neogenesis previously reported in the lungs from which the RNA samples were obtained for this study (17, 31). Strikingly, in the chronic experiment, consumption of the DHA high diet provided early and long-lasting protective effects against cSiO_2_-induced gene expression. Exhaustion of DHA's protective effects by week 13 is likely attributable to the low clearance rate of cSiO_2_ from the lung and continual reentry into the aforementioned inflammation cycle. Nevertheless, it might be speculated that such exhaustion might not occur in the cases of transient lupus triggers, such as infections, drugs, UV light, and stress.

While only three out of eight mice in the single dose group fed the CON diet showed altered gene response 24 h after a single cSiO_2_ dose, the responders' transcriptomes closely matched those for all eight mice 24 h after four weekly cSiO_2_ treatments. Notably, IFN- and chemokine-related genes were among those most affected. The inconsistency of the former might have resulted because of slow and incomplete cSiO_2_ distribution to the lower lung airways of some mice at 1 d following a single intranasal dose (25). Nonetheless, dietary DHA similarly suppressed cSiO_2_-triggered gene responses, suggesting that supplementation with this fatty acid could influence some of the very earliest effects of the particle.

Type I IFNs (IFNs), particularly IFN-α, induce an assemblage of up to 2000 genes referred to as the “IFN signature” that is a hallmark of SLE and other autoimmune diseases (44). In SLE patients, levels of type I IFN and IFN-inducible genes in peripheral blood mononuclear cells (PBMCs) are elevated and correlate with disease severity (45–48). GWAS investigations have further established a linkage between genes associated with type I IFN production and human lupus (49–53). The nCounter module used here contained 36 of the 63 genes in the human IFN signature designed by Li et al. (54). cSiO_2_ induced two-thirds of these genes in the lung, and remarkably, all were suppressed by DHA supplementation. The IFN-related genes most highly affected by DHA in this study have been associated with human SLE, including *Irf7* (55, 56)*, Oas2* (57–60), *Ifi44* (60–63)*, Ifit1* (64)*, Ifit3* (64)*, Isg15* (65)*, Nrlc5* (66), and *Mx2* (67).

Consistent with our findings, cSiO_2_ induced a type 1 IFN response in C57Bl/6 mice within 1 week of instillation (68). Moreover, cSiO_2_ instillation induced accumulation of macrophages, neutrophils, and lymphocytes and marked expression of *Ifnb, Irf7*, and *Ccl2* in the lungs of 129SV mice, whereas these responses were significantly reduced in corresponding interferon α/β receptor knockout mice (69).

Also in agreement with our results here, preclinical studies suggest that type 1 interferons promote autoimmunity. For example, IFN-α administration to NZBWF1 mice quickened lupus onset (70, 71) and diminished the effectiveness of pharmacological interventions (4, 71). Furthermore, type I IFN overexpression hastened autoantibody production and autoimmune disease progression in NZBWF1 mice (71). Finally, type I IFN receptor deletion diminished autoantibody production and disease activity in NZBWF1 mice (72) and four other lupus-prone models (73–75). Together, these reports support our findings that the IFN signature was closely linked to cSiO_2_-induced autoimmune disease progression in NZBWF1 mice and, furthermore, that both the signature and disease were ablated by DHA supplementation.

Our observation that cSiO_2_ exposure altered IFN-related gene expression provides unique insight into putative early targets and mechanisms of action for the particle and how its effects are ameliorated by ω-3 fatty acids. A candidate cell type for the effects of cSiO_2_ and DHA is the plasmacytoid dendritic cell (pDC), a primary producer of IFN-α (76). pDC depletion in lupus-prone mice prior to disease initiation resulted in reduction in autoimmune pathology (77–79). Lupus-prone mice haplodeficient for a pDC-specific transcription factor contained fewer pDCs and exhibited reduced disease symptoms, particularly those related to germinal center development and autoantibody production (80). pDCs contain endosomal toll-like receptor (TLR)-7 and TLR-9 that recognize single-strand RNA and DNA, respectively (81–84). The IFNα-producing capacity of pDCs obtained from lupus patients was enhanced following TLR stimulation and these responses correlated with disease activity and serum IFN-α (85). Importantly, cSiO_2_ induced dsDNA release into the alveolar space in mice, and patients with silicosis had increased circulating dsDNA (68). RNA/DNA-containing immune complexes have been shown to elicit robust IFN-α production in pDCs (86–89). Indeed, prior studies have established that airway instillation of lupus-prone mice with cSiO_2_ triggers early and robust autoantibody responses to dsDNA, nuclear antigens, and histones coupled with increases in circulating immune complexes (11, 12, 15, 16). Thus, further investigation is needed to determine how cSiO_2_ affects pDC activation and type 1 IFN release and, furthermore, how DHA supplementation impairs this process.

Both type 1 IFNs and pDCs are therapeutic targets for SLE. Randomized, double-blind, placebo-controlled phase IIb clinical trials have suggested the efficacies of sifalimumab, an anti-IFNα monoclonal antibody (90) and anifrolumab, a type I interferon (IFN) receptor antagonist (91, 92), for treating moderate-to-severe SLE. Very recently, a large double-blind, placebo-controlled phase 3 clinical trial (TULIP-2) was completed that reported that intravenous anifrolumab was superior to placebo for multiple efficacy endpoints, including overall disease activity, skin disease, and oral corticosteroid tapering (93). Blood dendritic cell antigen 2 (BDCA2), a pDC specific receptor, has been targeted for preclinical and clinical investigation of lupus treatment (94). In non-human primates, anti-BDCA2 antibodies suppress both IFNα-production by pDCs (95). Recently, it was reported that the humanized anti-BDCA2 antibody suppressed the IFN signature and ameliorated cutaneous lesions in human lupus patients (96).

DHA's capacity to ameliorate cSiO_2_-upregulation of chemokine genes is also remarkable. Affected genes included chemokine ligands/receptors with C-X-C motif including *Cxcl3, Cxcl9, Cxcl10, Cxcl12, Cxcl13, Cxcr1*, and *Cxcr3*. Of particular relevance, Cxcl13 (a.k.a. B-lymphocyte chemoattractant [BLC]), is preferentially produced by follicular dendritic cells in B-cell follicles of lymphoid organs (97), a population that is upregulated in the lungs by cSiO_2_ (31). Treatment with anti-CXCL13 antibodies mitigated disease in murine models of autoimmune disease (98). Recently, Denton et al. (99) demonstrated in C57BL/6 mice that type I IFN produced after influenza infection induced CXCL13 expression in a lung fibroblasts, driving recruitment of B cells and initiating ectopic germinal center formation. Thus, type I IFN induces CXCL13, which, in combination with other stimuli, could provide the requisite stimuli to promote ELS. CXCL9 and CXCL10 share the receptor CXCR3 and are also induced by IFN (100). These chemokines direct activated T cell and natural killer cell migration.

Of further note, DHA mitigated cSiO_2_-driven upregulation of mRNAs for C-C-L motif ligands and their receptors (*Ccl2, Ccl7, Ccl8, Ccl12, Ccl20 Ccr2, Ccr5, Ccr6*). CCL2 (also known as monocyte chemoattractant protein 1 [MCP-1]) stimulates monocyte trafficking by binding to CCR2, and it is produced by mononuclear phagocytes, endothelial, and smooth muscle cells (101). cSiO_2_ exposure promoted MCP-1 elevation in BALF and plasma (15). Importantly, elevated plasma MCP-1 has been associated with increased disease severity in lupus patients (102, 103). CCL7 (MCP-3), CCL8 (MCP-2), and CCL12 (MCP-5) are structurally related and share properties with CCL2. Finally, CCR6 and its ligand CCL20 (MCP-3α) coordinate regulation of effective humoral responses also have been linked to autoantibody-driven autoimmune diseases including lupus (104).

Our prior histological assessment (33) of the kidneys employed in this study indicated that 13 weeks after cSiO_2_ instillation, CON-fed mice exhibited proteinuria with moderate to severe diffuse glomerulonephritis. Consistent with this observation, we found here that there was extensive upregulation of immune genes in kidney tissue, most notably those associated with IFN, chemokines, antigen presentation, and MHC expression. Mice fed DHA exhibited marked reduction of these histopathological lesions reflecting the dramatic suppression of massive cell recruitment and gene expression during cSiO_2_-driven inflammation. Since cSiO_2_ is retained the lung and its associated lymph nodes (41, 42), the cellular and gene responses in the kidney most likely result from autoantibodies and immune complexes originating in the lung. We speculate that these travel via the systemic compartment and consequently deposit in the kidney evoking vigorous inflammation and ultimately glomerulonephritis. Accordingly, the kidney histological and mRNA profiles very likely were an outcome of cSiO_2_-triggered ELS formation in the lung. Since DHA supplementation impeded pulmonary ectopic lymphoid neogenesis in the lung, it follows that DHA also prevented downstream cell recruitment and gene expression in the kidney. Finally, it should be noted that gene responses in the spleen to cSiO_2_ and DHA treatments were very modest compared to the lung and kidney. This result may be expected because the spleen contains many more non-activated cells than lung which would dilute expression of immune genes.

Our finding that DHA supplementation impeded genes associated with lupus flaring and glomerulonephritis are consistent with several clinical trials suggesting that there are potential benefits of ω-3 intake by SLE patients. To date, nine controlled clinical studies have tested ω-3-containing fish oil supplements on lupus. Supplementation duration varied from 10 to 52 weeks, and patients per trial ranged from 12 to 85 subjects. ω-3 intake ranged from 0.54 to 3.60 g/d EPA and 0.30 to 2.25 g/d DHA. Five investigations showed ω-3 supplementation modulated and improved SLE scores (105–109). Another trial included both non-nephritic SLE patients and lupus nephritis patients and found significant improvements in several SLE markers in blood (110). One study reported improvement in clinical parameters after 3 months but not at 6 months (111). In contrast, two other clinical studies reported no therapeutic benefits of ω-3 for patients with SLE (112) or lupus nephritis (113). General limitations of the clinical studies run to date include low numbers of patients, short study length, insufficient ω-3 dosage, lack of corroborating fatty acid analyses, and/or not controlling impact of concurrent SLE therapies. It should be noted that the clinical studies to date have typically used between 1 to 5 g of ω-3 mixtures of DHA plus EPA. The observation that diets providing human energy equivalents of 5 g/d DHA elicited more marked and longer lasting effects than the DHA low diet is potentially a critical consideration for future clinical studies. Thus, additional studies are required to examine the potential differential effects of DHA and EPA.

## Conclusion

Taken together, the findings reported herein that DHA supplementation impeded IFN and chemokine gene expression associated with lupus flaring and nephritis supports the contention that dietary supplementation with ω-3 fatty acids may be a viable adjunct for the prevention and treatment of SLE. A potential mechanism linking dietary ω-3 supplementation to the observed transcriptional changes is the alteration of the cell membrane lipid profile, as DHA elevates membrane ω-3 HUFAs at the expense of ω-6 HUFAs. Consequently, this shift in membrane lipids could modify HUFA-derived metabolite profiles. Lipid metabolites derived from the ω-6 HUFA arachidonic acid (ARA) include the proinflammatory prostaglandins, leukotrienes, and thromboxanes. Alternatively, metabolites derived from ω-3 HUFAs, including DHA, docosapentaenoic acid (DPA) and EPA have been termed specialized pro-resolving mediators due to their capacity to resolve inflammatory responses. These mediators, as well as the free fatty acids from which they are metabolized, have been shown to participate in anti-inflammatory signaling pathways inhibiting the transcription of pro-inflammatory genes. We chose to assess levels of membrane ω-3 HUFAs as a percent of total HUFA (ω-3 HUFA score) (Figure 6), as defined by Lands and coworkers (19), to accentuate the competition between metabolism of ω-3 and ω-6 HUFAs. We found robust negative correlations between the ω-3 HUFA score and many of the gene pathways induced by cSiO_2_, providing strong evidence that incorporation into the phospholipid membrane is central to DHA's protective effects. Additional research is needed to determine how the ω-3 HUFA score and IFN signature could be used in a precision medicine approach to identify lupus patients that may benefit from ω-3 supplementation.

## Data Availability Statement

The data output from nSolver analyses for this study can be found at https://doi.org/10.26078/4697-1p77. The raw data supporting the conclusions of this manuscript will be made available by the authors, without undue reservation, to any qualified researcher.

## Ethics Statement

The animal study was reviewed and approved by Institutional Animal Care and Use Committee at Michigan State University (AUF #01/15-021-00).

## Author Contributions

AB: data analyses and interpretation, statistical analysis, figure preparation, and manuscript preparation and submission. MB: study design, animal study coordination, necropsy, RNA analysis, data analyses and interpretation, manuscript preparation, and project funding. PC: experimental design, immunohistochemistry, and cytokine analyses. KW: fatty acid analyses, data analyses, and manuscript preparation. KG: animal study coordination, RNA analysis, data analyses, and manuscript preparation. AH: experimental design, data interpretation, manuscript writing, and project funding. JH: study design, lung and kidney histopathology, morphometry, data analyses, manuscript preparation, and project funding. JP: planning, coordination, oversight, manuscript preparation and submission, and project funding.

### Conflict of Interest

The authors declare that the research was conducted in the absence of any commercial or financial relationships that could be construed as a potential conflict of interest.

## Supplementary Material

Presentation 1 File with Supplementary Figures 1–11.

The supplementary data files are available at: https://doi.org/10.26078/4697-1p77.

Supplementary File 1 Customized probe annotation file for the NanoString nCounter Mouse PanCancer Immune Profiling Panel.

Supplementary File 2 Microsoft Excel document with output from nSolver for differential expression analyses.

Supplementary File 3 Microsoft Excel document with output from nSolver for global and directed significance scores for immune pathways.

Supplementary File 4 Microsoft Excel document with output from nSolver for immune pathway Z scores for pairwise comparisons.

Supplementary File 5 Microsoft Excel document with protein networks obtained from STRING database and clusters predicted by the MCL algorithm.

## References

[1] Pons-Estel GJ Ugarte-Gil MF Alarcon GS. Epidemiology of systemic lupus erythematosus. Expert Rev Clin Immunol. (2017) 13:799–814. 10.1080/1744666X.2017.1327352 28471259

[2] Flores-Mendoza G Sanson SP Rodriguez-Castro S Crispin JC Rosetti F. Mechanisms of tissue injury in lupus nephritis. Trends Mol Med. (2018) 24:364–78. 10.1016/j.molmed.2018.02.003 29526595

[3] Sang A Yin Y Zheng YY Morel L. Animal models of molecular pathology systemic lupus erythematosus. Prog Mol Biol Transl Sci. (2012) 105:321–70. 10.1016/B978-0-12-394596-9.00010-X 22137436

[4] Liu Z Bethunaickan R Huang W Lodhi U Solano I Madaio MP Interferon alpha accelerates murine SLE in a T cell dependent manner. Arthr Rheum. (2011) 63:219–29. 10.1002/art.30087 20954185 PMC3014995

[5] Dai C Wang H Sung SS Sharma R Kannapell C Han W. Interferon alpha on NZM2328.Lc1R27: enhancing autoimmunity and immune complex-mediated glomerulonephritis without end stage renal failure. Clin Immunol. (2014) 154:66–71. 10.1016/j.clim.2014.06.008 24981059 PMC4167638

[6] Jacob N Guo S Mathian A Koss MN Gindea S Putterman C. B Cell and BAFF dependence of IFN-alpha-exaggerated disease in systemic lupus erythematosus-prone NZM 2328 mice. J Immunol. (2011) 186:4984–93. 10.4049/jimmunol.1000466 21383240 PMC3074466

[7] Ansel JC Mountz J Steinberg AD DeFabo E Green I. Effects of UV radiation on autoimmune strains of mice: increased mortality and accelerated autoimmunity in BXSB male mice. J Invest Dermatol. (1985) 85:181–6. 10.1111/1523-1747.ep12276652 3897390

[8] Wolf SJ Estadt SN Theros J Moore T Ellis J Liu J. Ultraviolet light induces increased T cell activation in lupus-prone mice via type I IFN-dependent inhibition of T regulatory cells. J Autoimmun. (2019) 103:102291. 10.1016/j.jaut.2019.06.002 31248690 PMC6708464

[9] Clark KL Reed TJ Wolf SJ Lowe L Hodgin JB Kahlenberg JM. Epidermal injury promotes nephritis flare in lupus-prone mice. J Autoimmun. (2015) 65:38–48. 10.1016/j.jaut.2015.08.005 26305061 PMC4679658

[10] Parks CG Miller FW Pollard KM Selmi C Germolec D Joyce K. Expert panel workshop consensus statement on the role of the environment in the development of autoimmune disease. Int J Mol Sci. (2014) 15:14269–97. 10.3390/ijms150814269 25196523 PMC4159850

[11] Brown JM Archer AI Pfau IC Holian A. Silica accelerated systemic autoimmune disease in lupus-prone New Zealand mixed mice. Clin Exp Immunol. (2003) 131:415–21. 10.1046/j.1365-2249.2003.02094.x 12605693 PMC1808650

[12] Brown JM Pfau JC Holian A. Immunoglobulin and lymphocyte responses following silica exposure in New Zealand mixed mice. Inhal Toxicol. (2004) 16:133–9. 10.1080/08958370490270936 15204774

[13] Brown JM Schwanke CM Pershouse MA Pfau JC Holian A. Effects of rottlerin on silica-exacerbated systemic autoimmune disease in New Zealand mixed mice. Am J Physiol Lung Cell Mol Physiol. (2005) 289:L990–8. 10.1152/ajplung.00078.2005 16040631

[14] Brown JM Swindle EJ Kushnir-Sukhov NM Holian A Metcalfe DD. Silica-directed mast cell activation is enhanced by scavenger receptors. Am J Respir Cell Mol Biol. (2007) 36:43–52. 10.1165/rcmb.2006-0197OC 16902192 PMC1899302

[15] Bates MA Brandenberger C Langohr I Kumagai K Harkema JR Holian A. Silica triggers inflammation and ectopic lymphoid neogenesis in the lungs in parallel with accelerated onset of systemic autoimmunity and glomerulonephritis in the lupus-prone NZBWF1 mouse. PLoS ONE. (2015) 10:e0125481. 10.1371/journal.pone.0125481 25978333 PMC4433215

[16] Bates MA Brandenberger C Langohr II Kumagai K Lock AL Harkema JR. Silica-triggered autoimmunity in lupus-prone mice blocked by docosahexaenoic acid consumption. PLoS ONE. (2016) 11:e0160622. 10.1371/journal.pone.0160622 27513935 PMC4981380

[17] Bates MA Benninghoff AD Gilley KN Holian A Harkema JR Pestka JJ. Mapping of dynamic transcriptome changes associated with silica-triggered autoimmune pathogenesis in the lupus-prone NZBWF1 mouse. Front Immunol. (2019) 10:632. 10.3389/fimmu.2019.00632 30984195 PMC6450439

[18] Calder PC. Omega-3 fatty acids and inflammatory processes: from molecules to man. Biochem Soc Trans. (2017) 45:1105–15. 10.1042/BST20160474 28900017

[19] Lands B Bibus D Stark KD. Dynamic interactions of n-3 and n-6 fatty acid nutrients. Prostaglandins Leukot Essent Fatty Acids. (2018) 136:15–21. 10.1016/j.plefa.2017.01.012 28189338

[20] Harris WS. The Omega-6:Omega-3 ratio: a critical appraisal and possible successor. Prostaglandins Leukot Essent Fatty Acids. (2018) 132:34–40. 10.1016/j.plefa.2018.03.003 29599053

[21] Adarme-Vega TC Thomas-Hall SR Schenk PM. Towards sustainable sources for omega-3 fatty acids production. Curr Opin Biotechnol. (2014) 26:14–8. 10.1016/j.copbio.2013.08.003 24607804

[22] Robinson DR Prickett JD Makoul GT Steinberg AD Colvin RB. Dietary fish oil reduces progression of established renal disease in (NZB x NZW)F1 mice and delays renal disease in BXSB and MRL/1 strains. Arthr Rheum. (1986) 29:539–46. 10.1002/art.1780290412 3707632

[23] Robinson DR Xu LL Tateno S Guo M Colvin RB. Suppression of autoimmune disease by dietary n-3 fatty acids. J Lipid Res. (1993) 34:1435–44. 8409774

[24] Lim BO Jolly CA Zaman K Fernandes G. Dietary (n-6) and (n-3) fatty acids and energy restriction modulate mesenteric lymph node lymphocyte function in autoimmune-prone (NZB × NZW)F1 mice. J Nutr. (2000) 130:1657–64. 10.1093/jn/130.7.1657 10867032

[25] Jolly CA Muthukumar A Avula CP Troyer D Fernandes G. Life span is prolonged in food-restricted autoimmune-prone (NZB x NZW)F(1) mice fed a diet enriched with (n-3) fatty acids. J Nutr. (2001) 131:2753–60. 10.1093/jn/131.10.2753 11584100

[26] Bhattacharya A Lawrence RA Krishnan A Zaman K Sun D Fernandes G. Effect of dietary n-3 and n-6 oils with and without food restriction on activity of antioxidant enzymes and lipid peroxidation in livers of cyclophosphamide treated autoimmune-prone NZB/W female mice. J Amer Coll Nutr. (2003) 22:388–99. 10.1080/07315724.2003.10719322 14559931

[27] Halade GV Rahman MM Bhattacharya A Barnes J Chandrasekar B Fernandes G. Docosahexaenoic acid-enriched fish oil attenuates kidney disease and prolongs median and maximal life span of autoimmune lupus-prone mice. J Immunol. (2010) 184:5280–6. 10.4049/jimmunol.0903282 20368275 PMC2952419

[28] Halade GV Williams PJ Veigas JM Barnes JL Fernandes G. Concentrated fish oil (Lovaza(R)) extends lifespan and attenuates kidney disease in lupus-prone short-lived (NZBxNZW)F1 mice. Exp Biol Med. (2013) 238:610–22. 10.1177/1535370213489485 23918873 PMC3970264

[29] Pestka JJ Vines LL Bates MA He K Langohr I. Comparative effects of n-3, n-6 and n-9 unsaturated fatty acid-rich diet consumption on lupus nephritis, autoantibody production and CD4+ T cell-related gene responses in the autoimmune NZBWF1 mouse. PLoS ONE. (2014) 9:e100255. 10.1371/journal.pone.0100255 24945254 PMC4063768

[30] Wierenga KA Harkema JR Pestka JJ. Lupus, silica, and dietary omega-3 fatty acid interventions. Toxicol Pathol. (2019). 10.1177/0192623319878398 31725357 PMC6910909

[31] Bates MA Akbari P Gilley KN Wagner JG Li N Kopec AK. Dietary docosahexaenoic acid prevents silica-induced development of pulmonary ectopic germinal centers and glomerulonephritis in the lupus-prone NZBWF1 mouse. Front Immunol. (2018) 9:2002. 10.3389/fimmu.2018.02002 30258439 PMC6143671

[32] Reeves PG Nielsen FH Fahey GC Jr. AIN-93 purified diets for laboratory rodents: final report of the American Institute of Nutrition ad hoc writing committee on the reformulation of the AIN-76A rodent diet. J Nutr. (1993) 123:1939–51. 10.1093/jn/123.11.1939 8229312

[33] Hulsen T de Vlieg J Alkema W. BioVenn - a web application for the comparison and visualization of biological lists using area-proportional Venn diagrams. BMC Genomics. (2008) 9:488. 10.1186/1471-2164-9-488 18925949 PMC2584113

[34] Oliveros JC Venny. An Interactive Tool for Comparing Lists With Venn's Diagrams. (2007). Available online at: http://bioinfogp.cnb.csic.es/tools/venny/index.html (cited January 6, 2019).

[35] Metsalu T Vilo J. ClustVis: a web tool for visualizing clustering of multivariate data using Principal Component Analysis and heatmap. Nucl Acids Res. (2015) 43:W566–70. 10.1093/nar/gkv468 25969447 PMC4489295

[36] Calder PC. Marine omega-3 fatty acids and inflammatory processes: effects, mechanisms and clinical relevance. Biochim Biophys Acta. (2015) 1851:469–84. 10.1016/j.bbalip.2014.08.010 25149823

[37] Rabolli V Lison D Huaux F. The complex cascade of cellular events governing inflammasome activation and IL-1beta processing in response to inhaled particles. Part Fibre Toxicol. (2016) 13:40. 10.1186/s12989-016-0150-8 27519871 PMC4983011

[38] Joshi GN Knecht DA. Silica phagocytosis causes apoptosis and necrosis by different temporal and molecular pathways in alveolar macrophages. Apoptosis. (2013) 18:271–85. 10.1007/s10495-012-0798-y 23329178

[39] Li Y Cao X Liu Y Zhao Y Herrmann M. Neutrophil extracellular traps formation and aggregation orchestrate induction and resolution of sterile crystal-mediated inflammation. Front Immunol. (2018) 9:1559. 10.3389/fimmu.2018.01559 30034398 PMC6043642

[40] Desai J Foresto-Neto O Honarpisheh M Steiger S Nakazawa D Popper B. Particles of different sizes and shapes induce neutrophil necroptosis followed by the release of neutrophil extracellular trap-like chromatin. Sci Rep. (2017) 7:15003. 10.1038/s41598-017-15106-0 29101355 PMC5670218

[41] Absher MP Hemenway DR Leslie KO Trombley L Vacek P. Intrathoracic distribution and transport of aerosolized silica in the rat. Exp Lung Res. (1992) 18:743–57. 10.3109/01902149209031705 1327732

[42] Vacek PM Hemenway DR Absher MP Goodwin GD. The translocation of inhaled silicon dioxide: an empirically derived compartmental model. Fund Appl Toxicol. (1991) 17:614–26. 10.1016/0272-0590(91)90211-L 1665463

[43] Kawasaki H. A review of the fate of inhaled α-quartz in the lungs of rats. Inhal Toxicol. (2019) 31:25–34. 10.1080/08958378.2019.1597218 30997849

[44] Bengtsson AA Ronnblom L. Role of interferons in SLE. Best Pract Res Clin Rheumatol. (2017) 31:415–28. 10.1016/j.berh.2017.10.003 29224681

[45] Hooks JJ Moutsopoulos HM Geis SA Stahl NI Decker JL Notkins AL. Immune interferon in the circulation of patients with autoimmune disease. N Engl J Med. (1979) 301:5–8. 10.1056/NEJM197907053010102 449915

[46] Bengtsson AA Sturfelt G Truedsson L Blomberg J Alm G Vallin H Activation of type I interferon system in systemic lupus erythematosus correlates with disease activity but not with antiretroviral antibodies. Lupus. (2000) 9:664–71. 10.1191/096120300674499064 11199920

[47] Crow MK Kirou KA Wohlgemuth J. Microarray analysis of interferon-regulated genes in SLE. Autoimmunity. (2003) 36:481–90. 10.1080/08916930310001625952 14984025

[48] Kirou KA Lee C George S Louca K Papagiannis IG Peterson MG. Coordinate overexpression of interferon-α-induced genes in systemic lupus erythematosus. Arthritis Rheum. (2004) 50:3958–67. 10.1002/art.20798 15593221

[49] Harley JB Alarcon-Riquelme ME Criswell LA Jacob CO Kimberly RP Moser KL. Genome-wide association scan in women with systemic lupus erythematosus identifies susceptibility variants in ITGAM, PXK, KIAA1542 and other loci. Nat Genet. (2008) 40:204–10. 10.1038/ng.81 18204446 PMC3712260

[50] Hom G Graham RR Modrek B Taylor KE Ortmann W Garnier S. Association of systemic lupus erythematosus with C8orf13-BLK and ITGAM-ITGAX. N Engl J Med. (2008) 358:900–9. 10.1056/NEJMoa0707865 18204098

[51] Shen N Fu Q Deng Y Qian X Zhao J Kaufman KM. Sex-specific association of X-linked Toll-like receptor 7 (TLR7) with male systemic lupus erythematosus. Proc Natl Acad Sci USA. (2010) 107:15838–43. 10.1073/pnas.1001337107 20733074 PMC2936646

[52] Joseph S George NI Green-Knox B Treadwell EL Word B Yim S. Epigenome-wide association study of peripheral blood mononuclear cells in systemic lupus erythematosus: Identifying DNA methylation signatures associated with interferon-related genes based on ethnicity and SLEDAI. J Autoimmun. (2019) 96:147–57. 10.1016/j.jaut.2018.09.007 30301579

[53] Hiramatsu S Watanabe KS Zeggar S Asano Y Miyawaki Y Yamamura Y. Regulation of Cathepsin E gene expression by the transcription factor Kaiso in MRL/lpr mice derived CD4+ T cells. Sci Rep. (2019) 9:3054. 10.1038/s41598-019-38809-y 30816218 PMC6395770

[54] Li Q-Z Zhou J Lian Y Zhang B Branch VK Carr-Johnson F. Interferon signature gene expression is correlated with autoantibody profiles in patients with incomplete lupus syndromes. Clin Exp Immunol. (2010) 159:281–91. 10.1111/j.1365-2249.2009.04057.x 19968664 PMC2819494

[55] Xu WD Zhang YJ Xu K Zhai Y Li BZ Pan HF. IRF7, a functional factor associates with systemic lupus erythematosus. Cytokine. (2012) 58:317–20. 10.1016/j.cyto.2012.03.003 22455868

[56] Kawasaki A Furukawa H Kondo Y Ito S Hayashi T Kusaoi M. Association of PHRF1-IRF7 region polymorphism with clinical manifestations of systemic lupus erythematosus in a Japanese population. Lupus. (2012) 21:890–5. 10.1177/0961203312439333 22433914

[57] Ye S Guo Q Tang JP Yang CD Shen N Chen SL. Could 2'5'-oligoadenylate synthetase isoforms be biomarkers to differentiate between disease flare and infection in lupus patients? A pilot study. Clin Rheumatol. (2007) 26:186–90. 10.1007/s10067-006-0260-z 16565890

[58] Tang J Gu Y Zhang M Ye S Chen X Guo Q. Increased expression of the type I interferon-inducible gene, lymphocyte antigen 6 complex locus E, in peripheral blood cells is predictive of lupus activity in a large cohort of Chinese lupus patients. Lupus. (2008) 17:805–13. 10.1177/0961203308089694 18755862

[59] Grammatikos AP Kyttaris VC Kis-Toth K Fitzgerald LM Devlin A Finnell MD. A T cell gene expression panel for the diagnosis and monitoring of disease activity in patients with systemic lupus erythematosus. Clin Immunol. (2014) 150:192–200. 10.1016/j.clim.2013.12.002 24434273 PMC3932542

[60] Bing PF Xia W Wang L Zhang YH Lei SF Deng FY. Common marker genes identified from various sample types for systemic lupus erythematosus. PLoS ONE. (2016) 11:e0156234. 10.1371/journal.pone.0156234 27257790 PMC4892593

[61] Rodriguez-Carrio J Lopez P Alperi-Lopez M Caminal-Montero L Ballina-Garcia FJ Suarez A. IRF4 and IRGs delineate clinically relevant gene expression signatures in systemic lupus erythematosus and rheumatoid arthritis. Front Immunol. (2018) 9:3085. 10.3389/fimmu.2018.03085 30666255 PMC6330328

[62] Ulff-Moller CJ Asmar F Liu Y Svendsen AJ Busato F Gronbaek K. Twin DNA methylation profiling reveals flare-dependent interferon signature and b cell promoter hypermethylation in systemic lupus erythematosus. Arthr Rheum. (2018) 70:878–90. 10.1002/art.40422 29361205

[63] Fan H Zhao G Ren D Liu F Dong G Hou Y. Gender differences of B cell signature related to estrogen-induced IFI44L/BAFF in systemic lupus erythematosus. Immunol Lett. (2017) 181:71–8. 10.1016/j.imlet.2016.12.002 27923569

[64] Coit P Jeffries M Altorok N Dozmorov MG Koelsch KA Wren JD. Genome-wide DNA methylation study suggests epigenetic accessibility and transcriptional poising of interferon-regulated genes in naive CD4+ T cells from lupus patients. J Autoimmun. (2013) 43:78–84. 10.1016/j.jaut.2013.04.003 23623029 PMC3790645

[65] Yuan Y Ma H Ye Z Jing W Jiang Z. Interferon-stimulated gene 15 expression in systemic lupus erythematosus: diagnostic value and association with lymphocytopenia. Z Rheumatol. (2018) 77:256–62. 10.1007/s00393-017-0274-8 28204879

[66] Yeung KS Chung BH Choufani S Mok MY Wong WL Mak CC. Genome-wide DNA methylation analysis of chinese patients with systemic lupus erythematosus identified hypomethylation in genes related to the Type I interferon pathway. PLoS ONE. (2017) 12:e0169553. 10.1371/journal.pone.0169553 28085900 PMC5234836

[67] Wu C Zhao Y Lin Y Yang X Yan M Min Y. Bioinformatics analysis of differentially expressed gene profiles associated with systemic lupus erythematosus. Mol Med Rep. (2018) 17:3591–8. 10.3892/mmr.2017.8293 29257335 PMC5802164

[68] Benmerzoug S Rose S Bounab B Gosset D Duneau L Chenuet P. STING-dependent sensing of self-DNA drives silica-induced lung inflammation. Nat Commun. (2018) 9:5226. 10.1038/s41467-018-07425-1 30523277 PMC6283886

[69] Giordano G van den Brule S Lo Re S Triqueneaux P Uwambayinema F Yakoub Y. Type I interferon signaling contributes to chronic inflammation in a murine model of silicosis. Toxicol Sci. (2010) 116:682–92. 10.1093/toxsci/kfq158 20513754

[70] Liu Z Bethunaickan R Huang W Ramanujam M Madaio MP Davidson A IFNα confers resistance of SLE nephritis to therapy in NZB/WF1 mice. J Immunol. (2011) 187:1506–13. 10.4049/jimmunol.1004142 21705616 PMC3140572

[71] Mathian A Weinberg A Gallegos M Banchereau J Koutouzov S IFN-α induces early lethal lupus in preautoimmune (New Zealand Black x New Zealand White) F1 but not in BALB/c mice. J Immunol. (2005) 174:2499–506. 10.4049/jimmunol.174.5.2499 15728455

[72] Santiago-Raber ML Baccala R Haraldsson KM Choubey D Stewart TA Kono DH. Type-I interferon receptor deficiency reduces lupus-like disease in NZB mice. J Exp Med. (2003) 197:777–88. 10.1084/jem.20021996 12642605 PMC2193854

[73] Braun D Geraldes P Demengeot J. Type I interferon controls the onset and severity of autoimmune manifestations in lpr mice. J Autoimmun. (2003) 20:15–25. 10.1016/S0896-8411(02)00109-9 12604309

[74] Jorgensen TN Roper E Thurman JM Marrack P Kotzin BL. Type I interferon signaling is involved in the spontaneous development of lupus-like disease in B6.Nba2 and (B6.Nba2 x NZW)F(1) mice. Genes Immun. (2007) 8:653–62. 10.1038/sj.gene.6364430 17882225

[75] Agrawal H Jacob N Carreras E Bajana S Putterman C Turner S. Deficiency of type I IFN receptor in lupus-prone New Zealand mixed 2328 mice decreases dendritic cell numbers and activation and protects from disease. J Immunol. (2009) 183:6021–9. 10.4049/jimmunol.0803872 19812195 PMC2766036

[76] Reizis B. plasmacytoid dendritic cells: development, regulation, and function. Immunity. (2019) 50:37–50. 10.1016/j.immuni.2018.12.027 30650380 PMC6342491

[77] Rowland SL Riggs JM Gilfillan S Bugatti M Vermi W Kolbeck R. Early, transient depletion of plasmacytoid dendritic cells ameliorates autoimmunity in a lupus model. J Exp Med. (2014) 211:1977–91. 10.1084/jem.20132620 25180065 PMC4172228

[78] Davison LM Jorgensen TN. Sialic acid-binding immunoglobulin-type lectin H-positive plasmacytoid dendritic cells drive spontaneous lupus-like disease development in B6.Nba2 mice. Arthritis Rheumatol. (2015) 67:1012–22. 10.1002/art.38989 25504931

[79] Takagi H Arimura K Uto T Fukaya T Nakamura T Choijookhuu N. Plasmacytoid dendritic cells orchestrate TLR7-mediated innate and adaptive immunity for the initiation of autoimmune inflammation. Sci Rep. (2016) 6:24477. 10.1038/srep24477 27075414 PMC4830934

[80] Sisirak V Ganguly D Lewis KL Couillault C Tanaka L Bolland S. Genetic evidence for the role of plasmacytoid dendritic cells in systemic lupus erythematosus. J Exp Med. (2014) 211:1969–76. 10.1084/jem.20132522 25180061 PMC4172218

[81] Cella M Jarrossay D Facchetti F Alebardi O Nakajima H Lanzavecchia A. Plasmacytoid monocytes migrate to inflamed lymph nodes and produce large amounts of type I interferon. Nat Med. (1999) 5:919–23. 10.1038/11360 10426316

[82] Siegal FP Kadowaki N Shodell M Fitzgerald-Bocarsly PA Shah K Ho S. The nature of the principal type 1 interferon-producing cells in human blood. Science. (1999) 284:1835–37. 10.1126/science.284.5421.1835 10364556

[83] Colonna M Trinchieri G Liu YJ. Plasmacytoid dendritic cells in immunity. Nat Immunol. (2004) 5:1219–26. 10.1038/ni1141 15549123

[84] Kawai T Akira S. The role of pattern-recognition receptors in innate immunity: update on Toll-like receptors. Nat Immunol. (2010) 11:373–84. 10.1038/ni.1863 20404851

[85] Murayama G Furusawa N Chiba A Yamaji K Tamura N Miyake S. Enhanced IFN-α production is associated with increased TLR7 retention in the lysosomes of palasmacytoid dendritic cells in systemic lupus erythematosus. Arthritis Res Ther. (2017) 19:234. 10.1186/s13075-017-1441-7 29052537 PMC5649081

[86] Lövgren T Eloranta ML Båve U Alm GV Rönnblom L. Induction of interferon-α production in plasmacytoid dendritic cells by immune complexes containing nucleic acid released by necrotic or late apoptotic cells and lupus IgG. Arthritis Rheum. (2004) 50:1861–72. 10.1002/art.20254 15188363

[87] Barrat FJ Meeker T Gregorio J Chan JH Uematsu S Akira S. Nucleic acids of mammalian origin can act as endogenous ligands for Toll-like receptors and may promote systemic lupus erythematosus. J Exp Med. (2005) 202:1131–9. 10.1084/jem.20050914 16230478 PMC2213213

[88] Means TK Latz E Hayashi F Murali MR Golenbock DT Luster AD. Human lupus autoantibody-DNA complexes activate DCs through cooperation of CD32 and TLR9. J Clin Invest. (2005) 115:407–17. 10.1172/JCI23025 15668740 PMC544604

[89] Lövgren T Eloranta ML Kastner B Wahren-Herlenius M Alm GV Rönnblom L. Induction of interferon-α by immune complexes or liposomes containing systemic lupus erythematosus autoantigen- and Sjögren's syndrome autoantigen-associated RNA. Arthritis Rheum. (2006) 54:1917–27. 10.1002/art.21893 16729300

[90] Khamashta M Merrill JT Werth VP Furie R Kalunian K Illei GG. Sifalimumab, an anti-interferon-alpha monoclonal antibody, in moderate to severe systemic lupus erythematosus: a randomised, double-blind, placebo-controlled study. Ann Rheum Dis. (2016) 75:1909–16. 10.1136/annrheumdis-2015-208562 27009916 PMC5099191

[91] Furie R Khamashta M Merrill JT Werth VP Kalunian K Brohawn P. Anifrolumab, an anti-interferon-alpha receptor monoclonal antibody, in moderate-to-severe systemic lupus erythematosus. Arthritis Rheumatol. (2017) 69:376–86. 10.1002/art.39962 28130918 PMC5299497

[92] Merrill JT Furie R Werth VP Khamashta M Drappa J Wang L. Anifrolumab effects on rash and arthritis: impact of the type I interferon gene signature in the phase IIb MUSE study in patients with systemic lupus erythematosus. Lupus Sci Med. (2018) 5:e000284. 10.1136/lupus-2018-000284 30588322 PMC6280909

[93] Morand EF Tanaka R Bruce Y Askanase A Richez C Bae S Ecacy and safety of anifrolumab in patients with moderate to severe systemic lupus erythematosus: results of the second phase 3 randomized controlled trial [abstract]. Arthr Rheumatol. (2019) 71(suppl 10). Available online at: https://acrabstracts.org/abstract/ecacy-and-safety-ofanifrolumab-in-patients-with-moderate-to-severe-systemic-lupus-erythematosus-results-of-thesecond-phase-3-randomized-controlled-trial/ (accessed December 2, 2019).

[94] Davison LM Jorgensen TN. New treatments for systemic lupus erythematosus on the horizon: targeting plasmacytoid dendritic cells to inhibit cytokine production. J Clin Cell Immunol. (2017) 8:534. 10.4172/2155-9899.1000534 29430334 PMC5804747

[95] Pellerin A Otero K Czerkowicz JM Kerns HM Shapiro RI Ranger AM. Anti-BDCA2 monoclonal antibody inhibits plasmacytoid dendritic cell activation through Fc-dependent and Fc-independent mechanisms. EMBO Mol Med. (2015) 7:464–76. 10.15252/emmm.201404719 25762615 PMC4403047

[96] Furie R Werth VP Merola JF Stevenson L Reynolds TL Naik H. Monoclonal antibody targeting BDCA2 ameliorates skin lesions in systemic lupus erythematosus. J Clin Invest. (2019) 129:359–71. 10.1172/JCI124466 30645203 PMC6391094

[97] Vermi W Lonardi S Bosisio D Uguccioni M Danelon G Pileri S. Identification of CXCL13 as a new marker for follicular dendritic cell sarcoma. J Pathol. (2008) 216:356–64. 10.1002/path.2420 18792075

[98] Klimatcheva E Pandina T Reilly C Torno S Bussler H Scrivens M. CXCL13 antibody for the treatment of autoimmune disorders. BMC Immunol. (2015) 16:6. 10.1186/s12865-015-0068-1 25879435 PMC4329654

[99] Denton AE Innocentin S Carr EJ Bradford BM Lafouresse F Mabbott NA. Type I interferon induces CXCL13 to support ectopic germinal center formation. J Exp Med. (2019) 216:621–37. 10.1084/jem.20181216 30723095 PMC6400543

[100] Metzemaekers M Vanheule V Janssens R Struyf S Proost P. Overview of the mechanisms that may contribute to the non-redundant activities of interferon-inducible CXC chemokine receptor 3 ligands. Front Immunol. (2017) 8:1970. 10.3389/fimmu.2017.01970 29379506 PMC5775283

[101] Palomino DC Marti LC. Chemokines and immunity. Einstein. (2015) 13:469–73. 10.1590/S1679-45082015RB3438 26466066 PMC4943798

[102] Bauer JW Petri M Batliwalla FM Koeuth T Wilson J Slattery C. Interferon-regulated chemokines as biomarkers of systemic lupus erythematosus disease activity: a validation study. Arthritis Rheum. (2009) 60:3098–107. 10.1002/art.24803 19790071 PMC2842939

[103] El-Shehaby A Darweesh H El-Khatib M Momtaz M Marzouk S El-Shaarawy N. Correlations of urinary biomarkers, TNF-like weak inducer of apoptosis (TWEAK), osteoprotegerin (OPG), monocyte chemoattractant protein-1 (MCP-1), and IL-8 with lupus nephritis. J Clin Immunol. (2011) 31:848–56. 10.1007/s10875-011-9555-1 21691937

[104] Lee AYS Korner H. The CCR6-CCL20 axis in humoral immunity and T-B cell immunobiology. Immunobiol. (2019) 224:449–454. 10.1016/j.imbio.2019.01.005 30772094

[105] Arriens C Hynan LS Lerman RH Karp DR Mohan C. Placebo-controlled randomized clinical trial of fish oil's impact on fatigue, quality of life, and disease activity in systemic lupus erythematosus. Nutr J. (2015) 14:82. 10.1186/s12937-015-0068-2 26283629 PMC4538741

[106] Lozovoy MAB Colado Simão AN Morimoto HK Scavuzzi BM Iriyoda TVM Reiche EMV. Fish oil n-3 fatty acids increase adiponectin and decrease leptin levels in patients with systemic lupus erythematosus. Marine Drugs. (2015) 13:1071–83. 10.3390/md13021071 25690094 PMC4344620

[107] Wright SA O'Prey FM McHenry MT Leahey WJ Devine AB Duffy EM. A randomised interventional trial of omega-3-polyunsaturated fatty acids on endothelial function and disease activity in systemic lupus erythematosus. Ann Rheum Dis. (2008) 67:841–8. 10.1136/ard.2007.077156 17875549

[108] Duffy EM Meenagh GK McMillan SA Strain JJ Hannigan BM Bell AL. The clinical effect of dietary supplementation with omega-3 fish oils and/or copper in systemic lupus erythematosus. J Rheumatol. (2004) 31:1551–6. 15290734

[109] Walton AJ Snaith ML Locniskar M Cumberland AG Morrow WJ Isenberg DA. Dietary fish oil and the severity of symptoms in patients with systemic lupus erythematosus. Ann Rheum Dis. (1991) 50:463–6. 10.1136/ard.50.7.463 1877851 PMC1004457

[110] Clark WF Parbtani A Naylor CD Levinton CM Muirhead N Spanner E. Fish oil in lupus nephritis: clinical findings and methodological implications. Kidney Int. (1993) 44:75–86. 10.1038/ki.1993.215 8355469

[111] Westberg G Tarkowski A. Effect of MaxEPA in patients with SLE. A double-blind, crossover study. Scand J Rheumatol. (1990) 19:137–43. 10.3109/03009749009102117 2186476

[112] Bello KJ Fang H Fazeli P Bolad W Corretti M Magder LS. Omega-3 in SLE: a double-blind, placebo-controlled randomized clinical trial of endothelial dysfunction and disease activity in systemic lupus erythematosus. Rheumatol Int. (2013) 33:2789–96. 10.1007/s00296-013-2811-3 23817872 PMC3805738

[113] Clark WF Parbtani A Huff MW Reid B Holub BJ Falardeau P. Omega-3 fatty acid dietary supplementation in systemic lupus erythematosus. Kidney Int. (1989) 36:653–60. 10.1038/ki.1989.242 2811063

