# Supplementary material for: Docosahexaenoic Acid Consumption Impedes Early Interferon- and Chemokine-Related Gene Expression While Suppressing Silica-Triggered Flaring of Murine Lupus
Source: Front Immunol. 2019 Dec 13;10:2851. doi: 10.3389/fimmu.2019.02851 (PMC6923248; doi:10.3389/fimmu.2019.02851)
Supplement: Presentation 1 — File with Supplementary Figures 1–11. [file Presentation_1.pdf]

frontiers in Immunology

Supplementary Material

Docosahexaenoic Acid Consumption Impedes Chemokine and Interferon-related Gene Expression Associated With Silica-Triggered Flaring of Murine Lupus

Abby D. Benninghoff*, Melissa A. Bates, Preeti S. Chauhan, Kathryn A. Wierenga, Kristen N. Gilley, Andrij Holian, Jack R. Harkema, and James J. Pestka*

*** Correspondence:**
Abby D. Benninghoff, Department of Animal, Dairy and Veterinary Sciences, Utah State University, 4815 Old Main Hill, Logan, UT, USA. E-Mail: abby.benninghoff@usu.edu

James J. Pestka, Department of Food Science and Human Nutrition, Michigan State University, East Lansing, MI 48824, U.S. E-mail: pestka@msu.edu

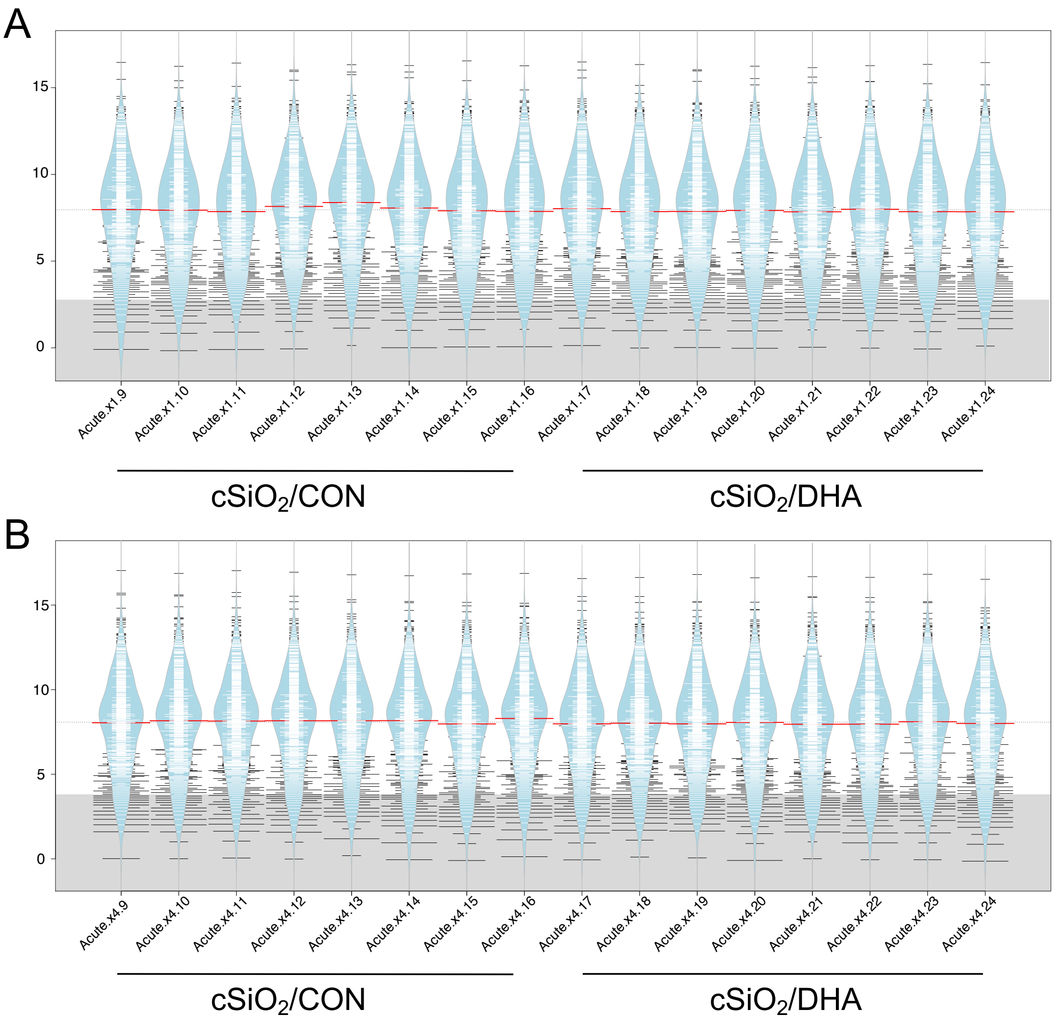

Supplementary Figure 1. Beanplots depicting distribution of normalized expression of NanoString PanCancer Immune gene panel – acute response in lung (experiments 1 and 2). Samples are named by dosing regimen (Acute.1x, single instillation; Acute.4x, four weekly instillations) and sample numbers, for which numbers 9-16 correspond to $cSiO_2$-exposed mice fed control diet; and 17-24, $cSiO_2$-exposed mice fed DHA diet. Red lines show the medians, white/black lines represent individual data points (line width indicates number of genes at that expression level), and blue polygons represent the estimated density of the data. The gray shaded region indicates genes excluded because of low signal. Plots were generated using BoxPlotR (shiny.chemgrid.org/boxplotr/). See Bates, et al.[1] for normalization data for vehicle-exposed, control-fed mice which were used for calculating $\log_2$ ratios, but not included in differential gene expression analysis for this manuscript.

[1] Bates MA, Benninghoff AD, Gilley KN, Holian A, Harkema JR, Pestka JJ. (2019) Mapping of dynamic transcriptome changes associated with silica-triggered autoimmune pathogenesis in the lupus-prone NZBWF1 mouse. *Frontiers in Immunology* (10):article 632. DOI: 10.3389/fimmu.2019.00632

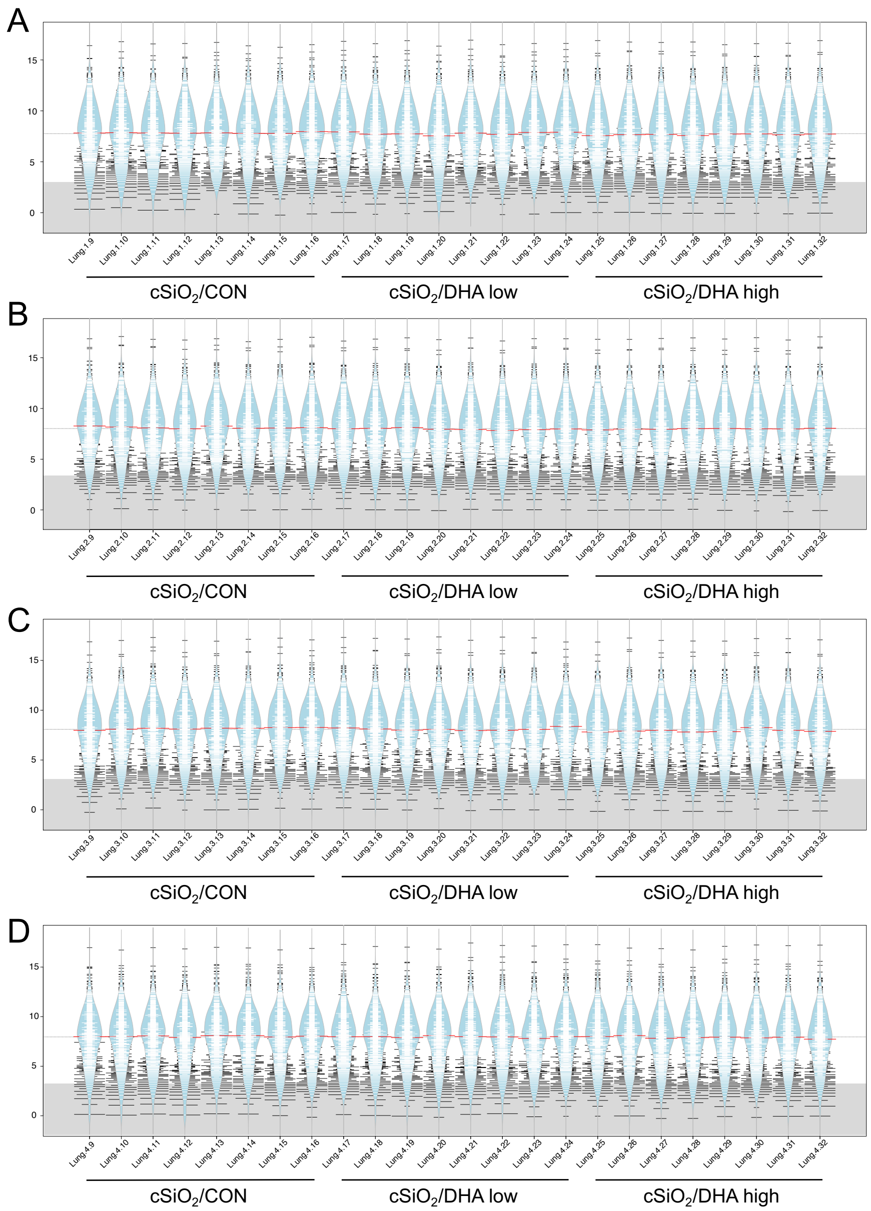

Supplementary Figure 2. Beanplots depicting distribution of normalized expression of NanoString PanCancer Immune gene panel – acute response in lung (experiments 1 and 2). Samples are named by "tissue.cohort.ID". Cohorts 1, 2, 3, and 4 correspond to samples obtained 1 (A), 5 (B), 9 (C), or 13 weeks (D) post instillation with $cSiO_2$. Sample ID numbers 9-16 correspond to cSiO2-exposed mice fed control (CON) diet; 17-24, $cSiO_2$-exposed mice fed DHA low diet; and 25-32, $cSiO_2$-exposed mice fed DHA high diet. Red lines show the medians, white/black lines represent individual data points (line width indicates number of genes at that expression level), and blue polygons represent the estimated density of the data. The gray shaded region indicates genes excluded because of low signal. Plots were generated using BoxPlotR (shiny.chemgrid.org/boxplotr/). See Bates, et al.[2] for normalization data for vehicle-exposed, control-fed mice which were used for calculating $\log_2$ ratios, but not included in differential gene expression analysis for this manuscript.

[2] Bates MA, Benninghoff AD, Gilley KN, Holian A, Harkema JR, Pestka JJ. (2019) Mapping of dynamic transcriptome changes associated with silica-triggered autoimmune pathogenesis in the lupus-prone NZBWF1 mouse. *Frontiers in Immunology* (10):article 632. DOI: 10.3389/fimmu.2019.00632

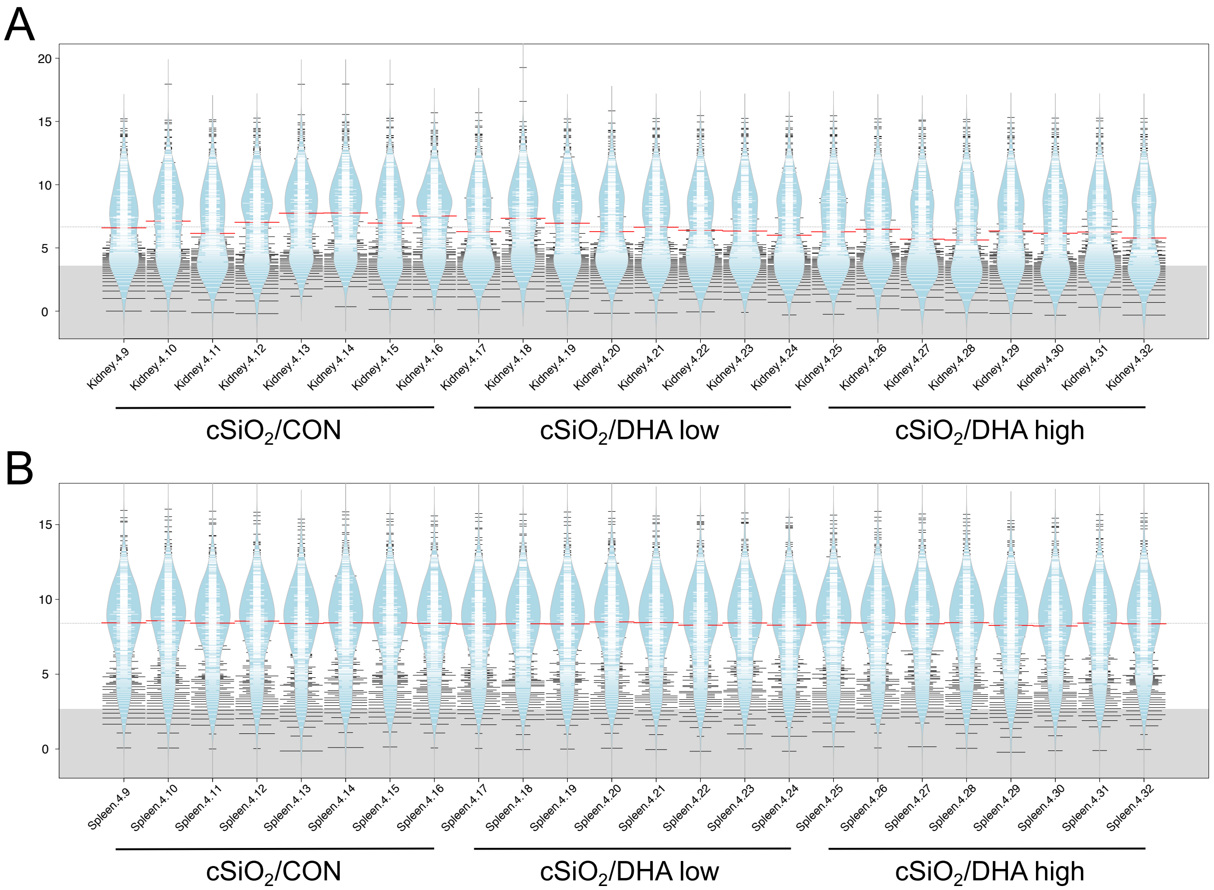

Supplementary Figure 3. Beanplots depicting distribution of normalized expression of NanoString PanCancer Immune gene panel – chronic response in kidney and spleen. Samples are named by "tissue.cohort.ID", with tissues kidney (A) and spleen (B) shown for the 4th cohort at 13 weeks post-instillation. ID numbers 9-16 correspond to cSiO2-exposed mice fed control (CON) diet; 17-24, $cSiO_2$-exposed mice fed DHA low diet; and 25-32, $cSiO_2$-exposed mice fed DHA high diet. Red lines show the medians, white/black lines represent individual data points (line width indicates number of genes at that expression level), and blue polygons represent the estimated density of the data. The shaded region indicates genes excluded because of low signal. Plots were generated using BoxPlotR (shiny.chemgrid.org/boxplotr/). See Bates, et al. [3] for normalization data for vehicle-exposed, control-fed mice which were used for calculating log_2 ratios, but not included in differential gene expression analysis for this manuscript.

[3] Bates MA, Benninghoff AD, Gilley KN, Holian A, Harkema JR, Pestka JJ. (2019) Mapping of dynamic transcriptome changes associated with silica-triggered autoimmune pathogenesis in the lupus-prone NZBWF1 mouse. *Frontiers in Immunology* (10):article 632. DOI: 10.3389/fimmu.2019.00632

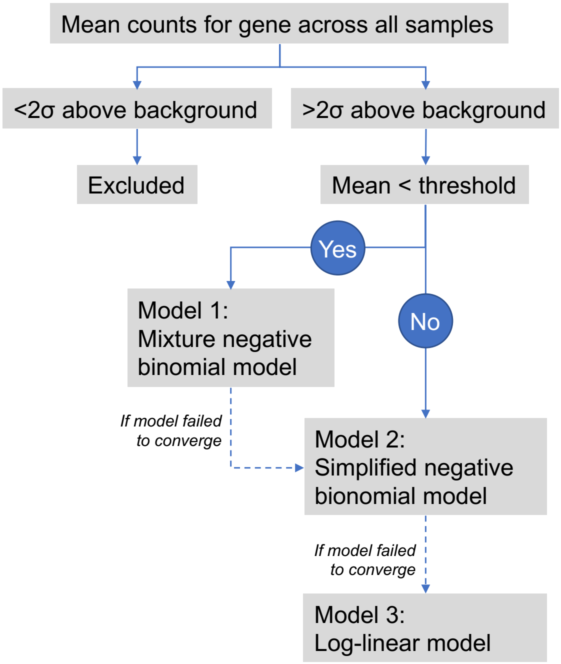

Supplementary Figure 4. Process flow for differential gene expression analysis using nSolver. As outlined by the supplied user manual[4], the mean of the gene across all samples is compared against the threshold, which is set as 10 times the background signal. If the gene mean is above the threshold, the mixture model in 1 (*MLE* function in R, Wald test to calculate a *p* value) is simplified to 2. If mixed model in 1 does not converge, the simplified model in 2 (*glm.nb* function in R/Mass) is applied instead. If model 2 does not converge, the log-linear model in 3 is used (*lm* function in R). For each gene, the optimal model applied for each gene is provided in Supplementary File 2 (model 1, "Wald"; model 2, "lm.nb"; model 3, "loglinear").

[4] NanoString Technologies, Inc. (2018) nCounter Advanced Analysis 2.0 Plugin for nSolver Software User Manual, vers. Jan 2018 (MAN-10030-03). Accessed at www.NanoString.com

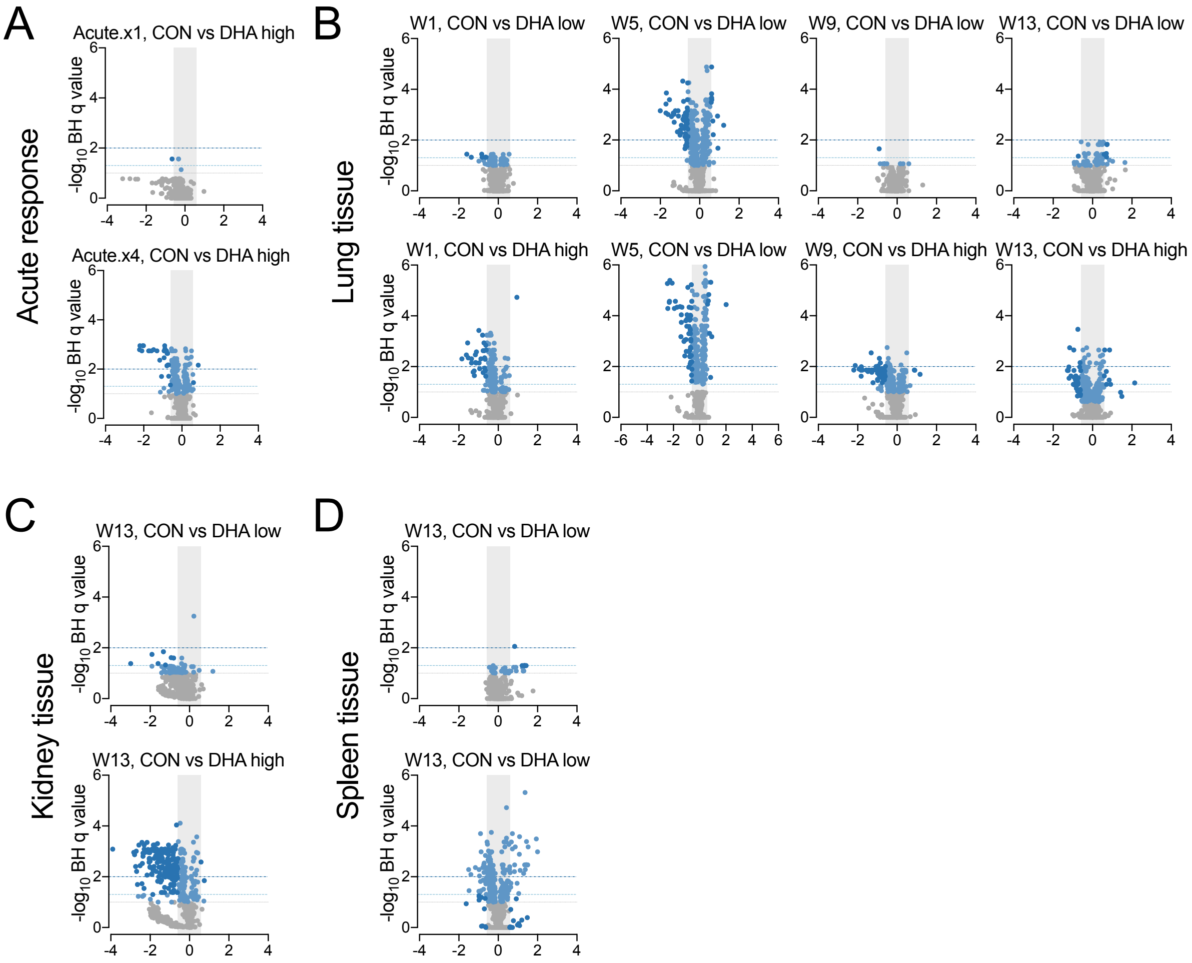

Supplementary Figure 5. Volcano plots depicting immune-related mRNA expression as measured using the NanoString Mouse PanCancer Immune Panel. Values shown are the $\log_2$ ratios of $cSiO_2$-treated mice fed either DHA low or DHA high diets with respect to non-supplemented controls (CON) controls plotted against the $-\log_{10}$ Benjamini-Hochberg FDR *q* value. A significant difference in gene expression was inferred for genes with BH *q* value <0.05 and $\log_2$ ratio >1 or <-1. Horizontal lines indicate *q* value cutoffs of 0.1, 0.05 and 0.01, and the vertical shaded region indicates expression values that do not meet the fold change cutoff for significance. (A) Lung tissues from mice fed DHA-supplemented diet versus dosing-matched CON diet control 1 day post single $cSiO_2$ instillation (Acute.x1) or 1 day post four weekly $cSiO_2$ instillations (Acute.x4). (B) Lung tissues from mice 1, 5, 9 or 13 weeks post $cSiO_2$ instillation fed either DHA low or DHA high supplemented diets compared to time-matched CON diet controls. (D) Kidney tissues from $cSiO_2$-treated (four weekly instillations) at 13 weeks post final instillation fed DHA low or DHA high supplemented diets compared to tissue-matched CON diet. (D) Spleen tissues from $cSiO_2$-treated (four weekly instillations) at 13 weeks post final instillation fed DHA low or DHA high supplemented diets compared to tissue-matched CON diet.

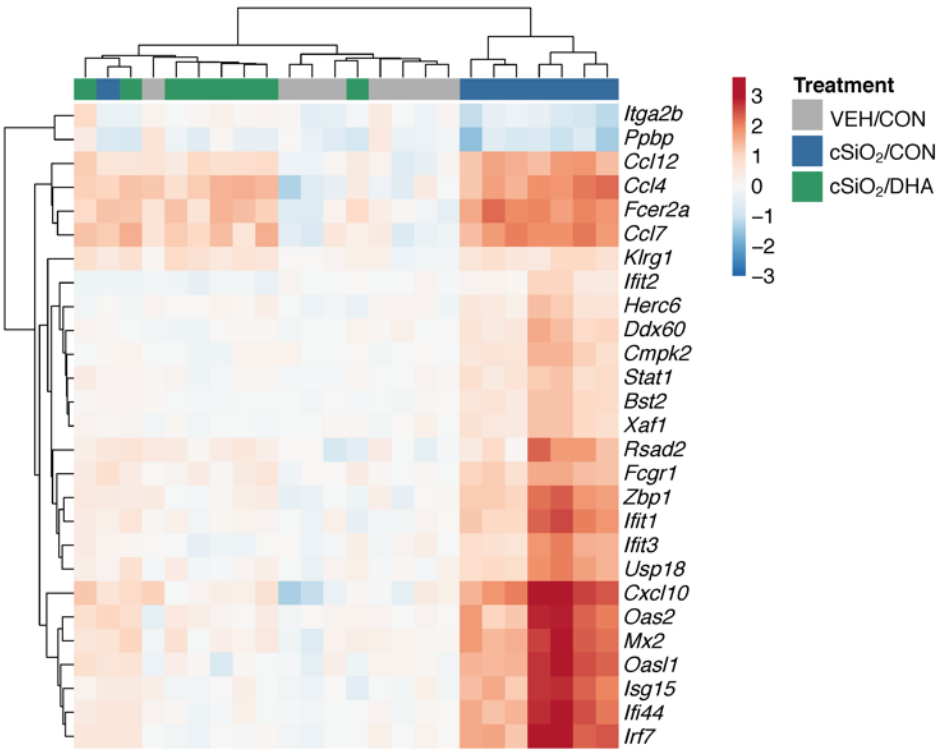

Supplementary Figure 6. Hierarchical cluster analysis of acute lung immune pathway transcriptome response in mice fed either CON or DHA-supplemented diets. Unsupervised, bidirectional hierarchical clustering was performed using the Euclidean distance method with average linkage. All genes significantly differentially regulated by DHA supplementation for either dosing protocol were included in the heatmap. The color scale represents the log_2 ratio versus vehicle-treated, CON-fed mice.

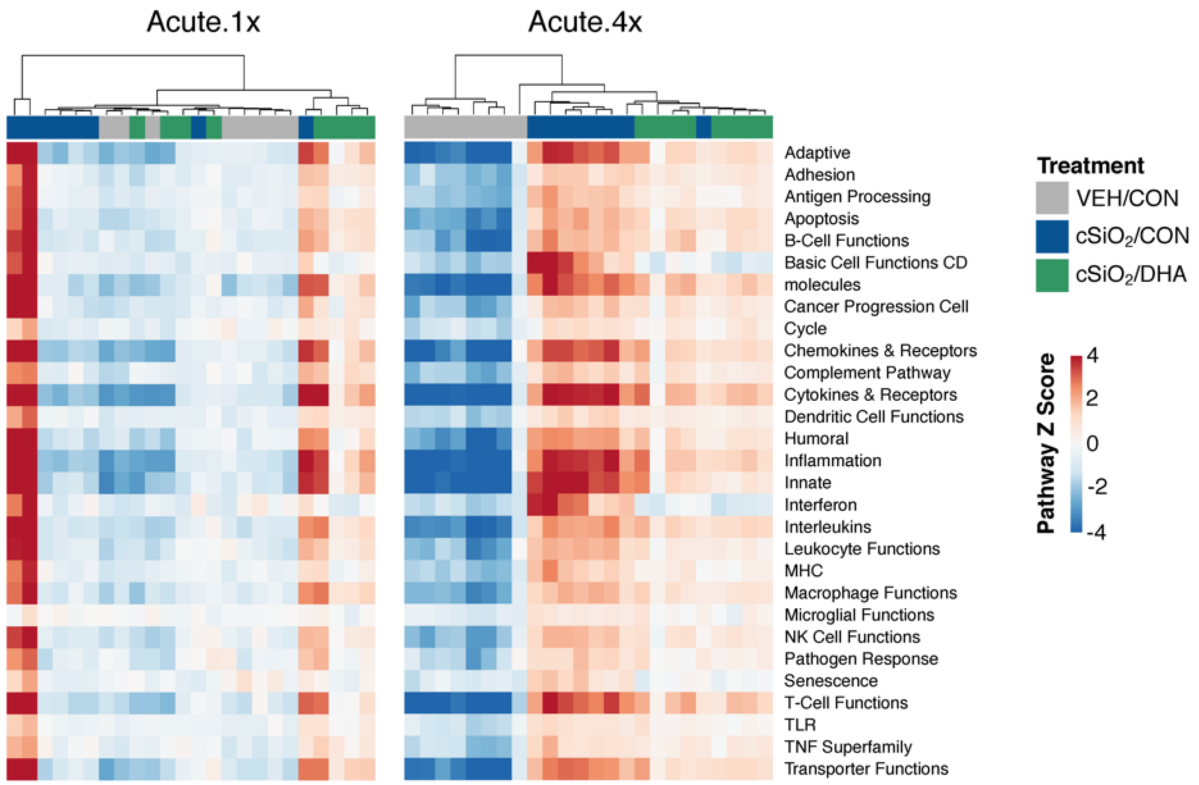

Supplementary Figure 7. Acute response individual pathway Z scores for immune pathways. Gene expression pathway scores were calculated as the first principal component of the pathway genes' normalized expression and standardized by Z scaling. Immune pathway Z scores are shown as a heat map organized via unidirectional hierarchical clustering (Euclidian distance method) by treatment/diet group for each dosing regimen (*n*=8) for all immune pathways captured in the NanoString PanCancer Immune Profiling gene set.

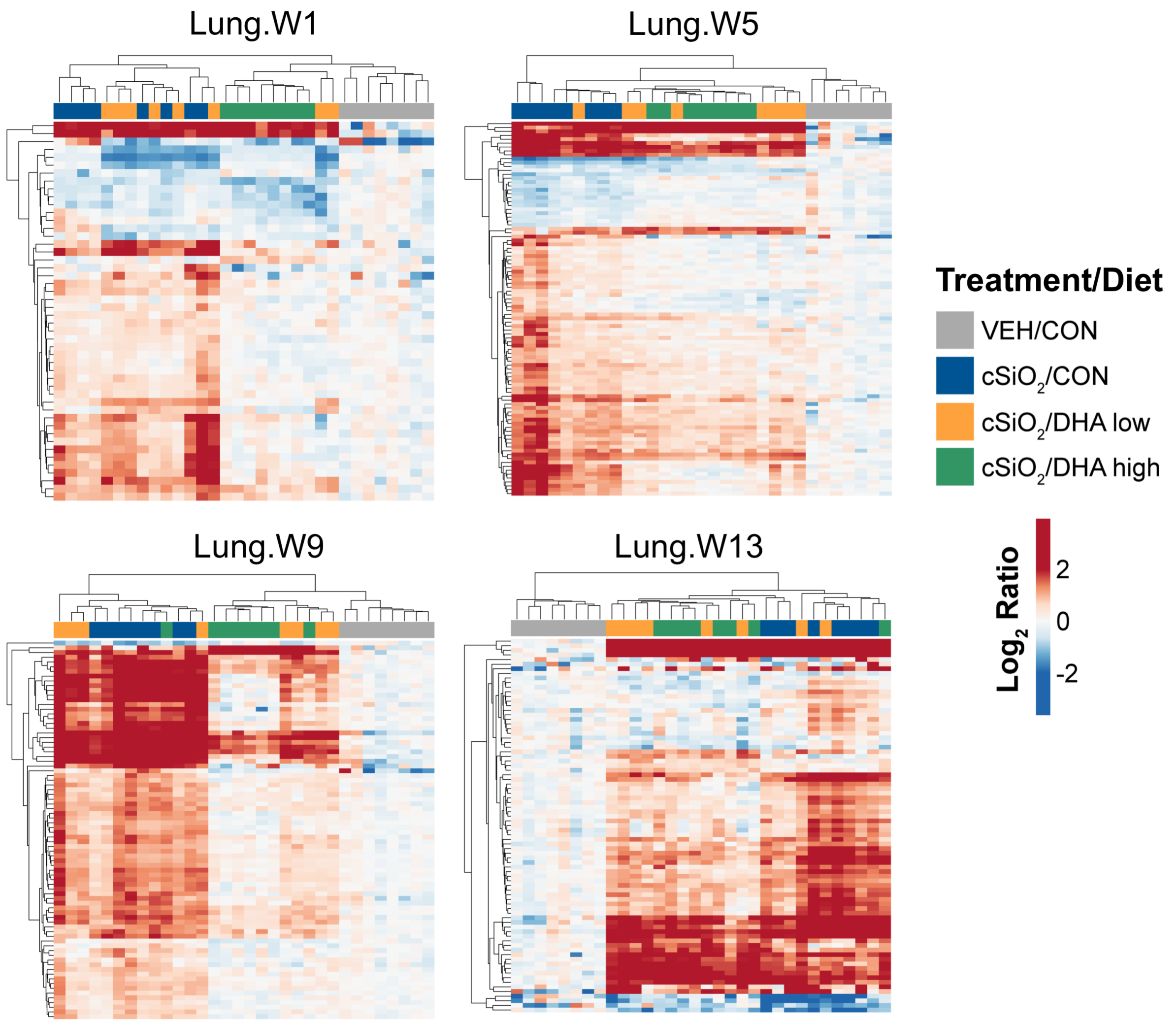

Supplementary Figure 8. Hierarchical cluster analyses of chronic lung immune pathway transcriptome response in mice fed either CON or DHA-supplemented diets. Unsupervised, bidirectional hierarchical clustering was performed using the Euclidean distance method with average linkage. For each time point, all genes differentially regulated by low or high DHA supplementation were included in the heatmap. The color scale represents the $\log_2$ ratio for each treatment versus vehicle (VEH)-treated, control (CON)-fed mice.

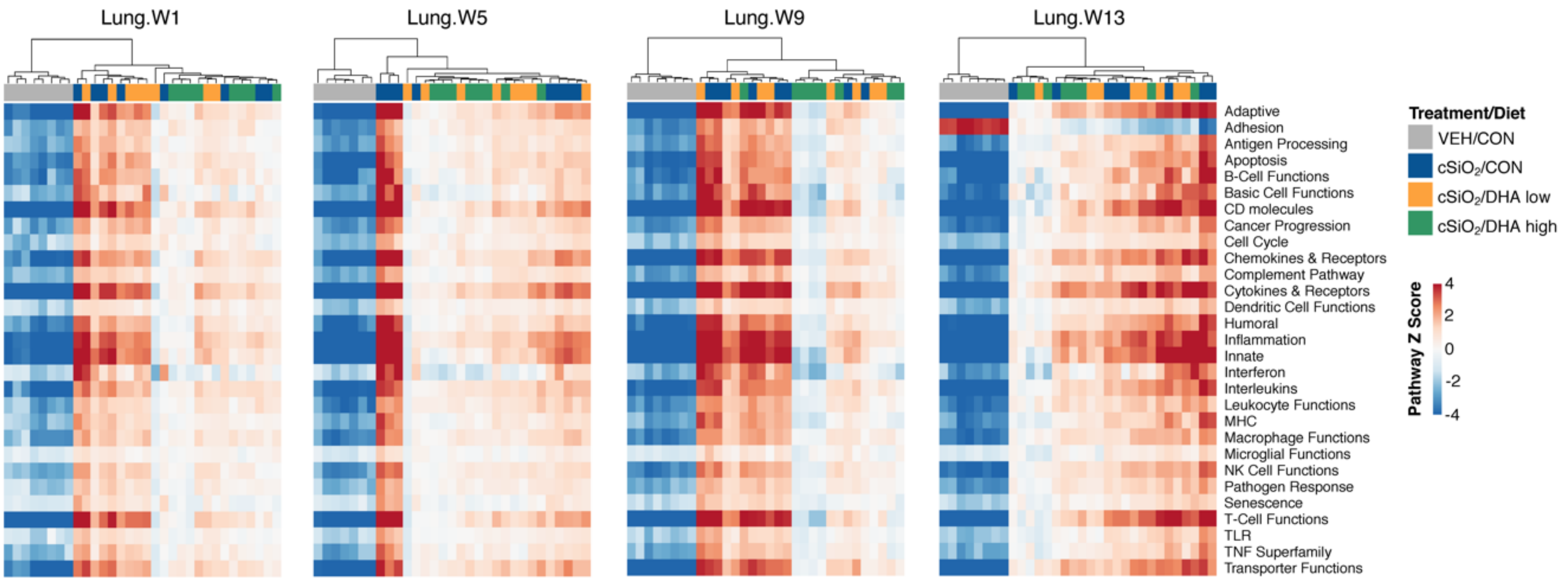

Supplementary Figure 9. Chronic response individual pathway Z scores for immune pathways. Gene expression pathway scores were calculated as the first principal component of the pathway genes' normalized expression and standardized by Z scaling. Immune pathway Z scores are shown as a heat map organized via unidirectional hierarchical clustering (Euclidian distance method) by treatment/diet group within each time point (*n*=7-8) for all immune pathways captured in the NanoString PanCancer Immune Profiling gene set.

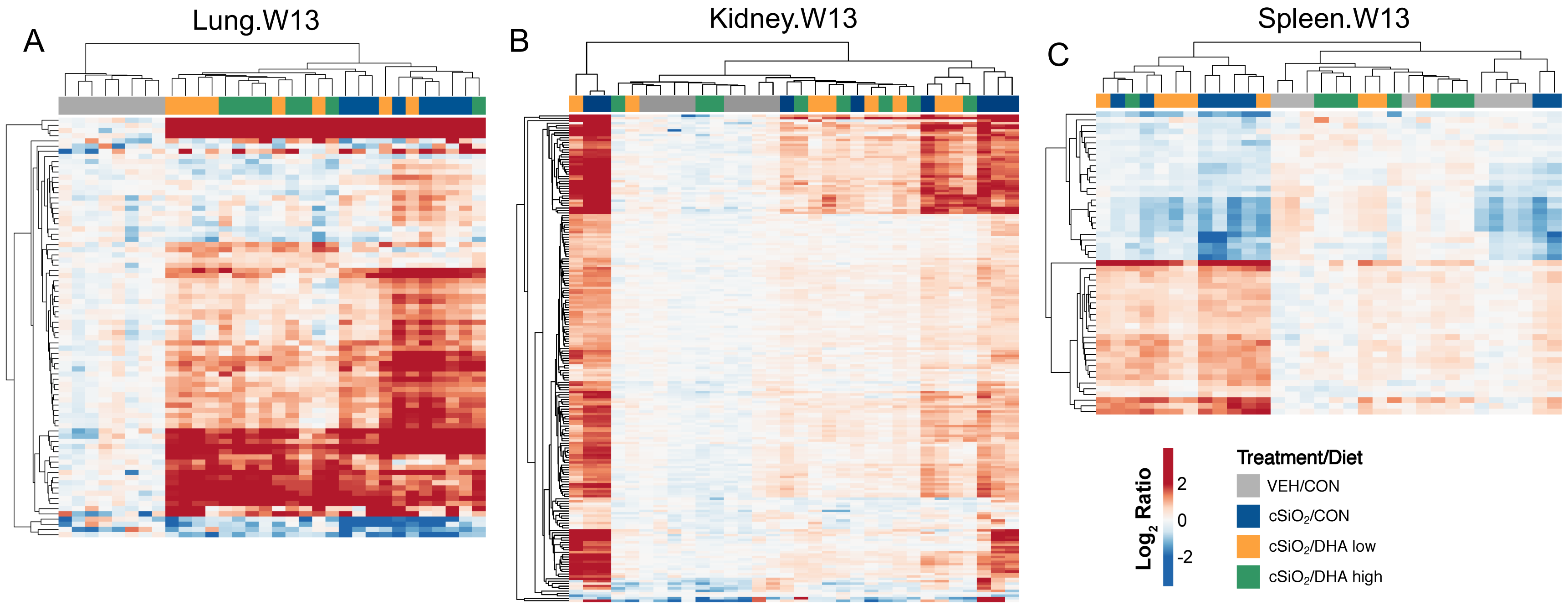

Supplementary Figure 10. Hierarchical cluster analysis for comparison of immune pathway transcriptome response in lung, kidney or spleen tissues of mice fed either CON or DHA-supplemented diets. Unsupervised, bidirectional hierarchical clustering was performed using the Euclidean distance method with average linkage. For each tissue, all genes differentially regulated by low or high DHA supplementation were included in the heatmap. The color scale represents the $\log_2$ ratio for each treatment versus vehicle (VEH)-treated, control (CON)-fed mice.

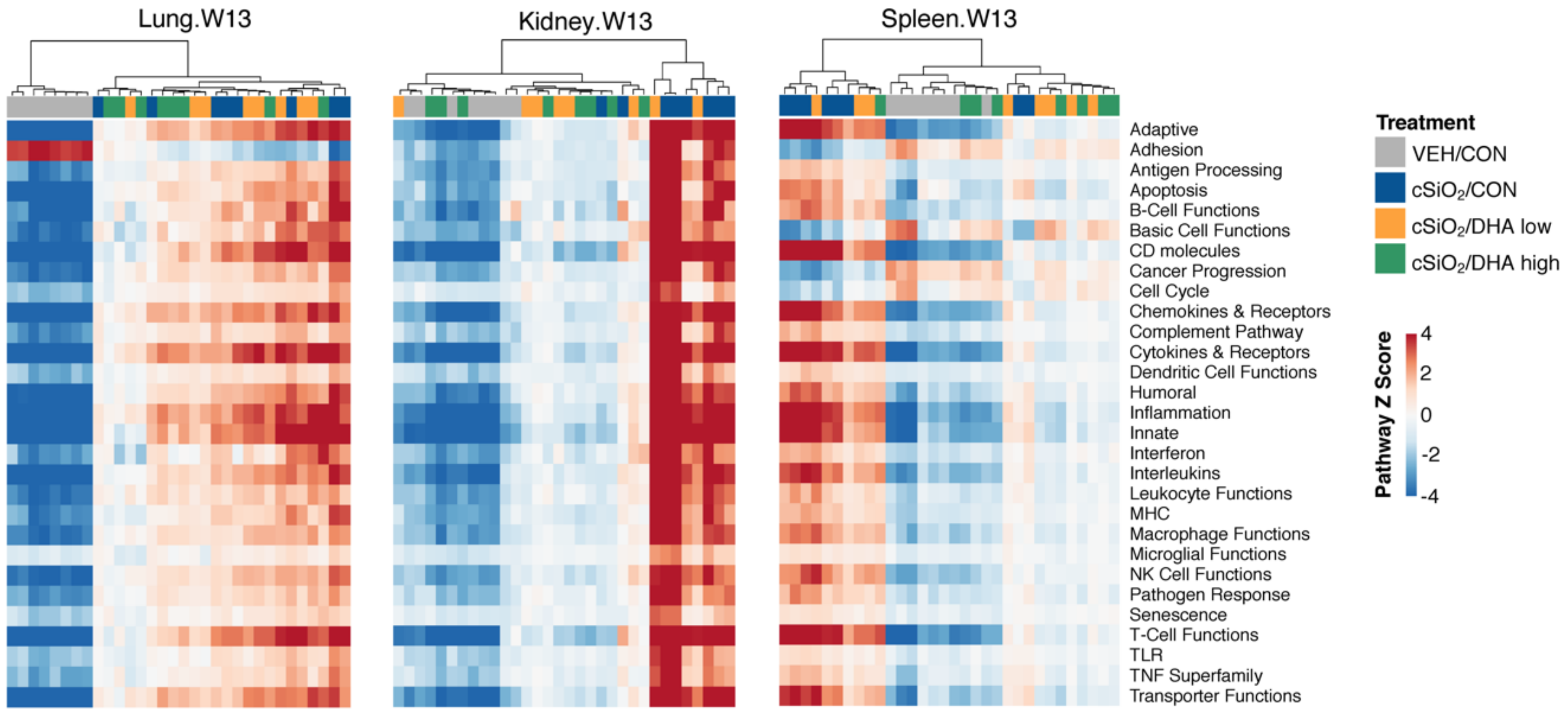

Supplementary Figure 11. Comparison of individual pathway Z scores for immune pathways in lung, kidney and spleen tissues. Gene expression pathway scores were calculated as the first principal component of the pathway genes' normalized expression and standardized by Z scaling. Immune pathway Z scores are shown as a heat map organized via unidirectional hierarchical clustering (Euclidian distance method) by treatment/diet group for each tissue (*n*=8) for all immune pathways captured in the NanoString PanCancer Immune Profiling gene set.
